# Nonlinear Ultrasound Crack Detection with Multi-Frequency Excitation—A Comparison

**DOI:** 10.3390/s21165368

**Published:** 2021-08-09

**Authors:** Frank Mevissen, Michele Meo

**Affiliations:** Research Centre, Department of Mechanical Engineering, University of Bath, Bath BA2 7AY, UK; f.mevissen@bath.ac.uk

**Keywords:** nonlinear ultrasound, modulation, crack detection, gas turbines

## Abstract

Nonlinear ultrasound crack detection methods are used as modern, non-destructive testing tools for inspecting early damages in various materials. Nonlinear ultrasonic wave modulation, where typically two or more frequencies are excited, was demonstrated to be a robust method for failure indicators when using measured harmonics and modulated response frequencies. The aim of this study is to address the capability of multi-frequency wave excitation, where more than two excitation frequencies are used, for better damage identification when compared to single and double excitation frequencies without the calculation of dispersion curves. The excitation frequencies were chosen in such a way that harmonic and modulated response frequencies meet at a specific frequency to amplify signal energy. A new concept of nonlinearity parameter grouping with multi-frequency excitation was developed as an early failure parameter. An analytical solution of the one-dimensional wave equation was derived with four fundamental frequencies, and a total of 64 individual and 30 group nonlinearity parameters. Experimental validation of the approach was conducted on metal plates with different types of cracks and on turbine blades where cracks originated under service conditions. The results showed that the use of multi-frequency excitation offers advantages in detecting cracks.

## 1. Introduction

Linear ultrasound techniques are proven testing methods for inspecting components. The failure detection entails measuring changes in elastic properties such as the speed of sound, attenuation, transmission coefficient, and reflection coefficient [1,2,3].

In 1964, Hikata et al. expanded this research and found that a sinusoidal ultrasonic wave distorts the fundamental frequency when it propagates in the presence of nonlinearities [4]. If an ultrasonic wave propagates into a solid body with the fundamental frequency and is disturbed during the propagation, harmonic frequencies are generated. When the amplitudes of these frequencies are measured and compared with the fundamental frequencies, these values represent an accurate indicator for detecting material changes [4]. When linear ultrasonic failure detection techniques reach their limits, nonlinear ultrasonic techniques revealed higher detection rates of incipient failures in materials [4,5].

The comparison of the amplitudes is conducted using derived nonlinearity parameters. Jhang et al. studied the second harmonic parameter *β* to explain its dependence on the wave shift, which enabled drawing conclusions about the material to be examined [6]. This work was extended by Frouin et al. [7], Rothenfusser et al. [8], and Yost et al. [9], who showed the direct dependence of the amplitudes of the fundamental frequency as well as the frequency of the second harmonic. The analytical derivations were conducted by Jeong et al. [10], while Ostrovky et al. investigated the nonlinearity parameters for geomaterials [11]. To determine the residual fatigue life of a component, Malfense Fierro and Meo developed the nonlinearity parameters for two superimposed frequencies [12,13,14], which was further developed by Jinpin et al. [15]. The third-order nonlinearity parameter with one driving frequency was derived from Amura and Meo [16]. The nonlinearity parameters of the second and third harmonic frequencies are shown in Equation (1) for one excitation frequency, where *A* is the amplitude of the fundamental frequency, *A_2_* is the amplitude of the second harmonic frequency, and *A_3_* is the amplitude of the third harmonic frequency:

(1) $$\begin{array}{c} \beta_{2f}\propto \frac{A_{2}}{A^{2}},\\ \gamma_{3f}\propto \frac{A_{3}}{A^{3}}. \end{array}$$

The excitation with two different frequencies up to third-order nonlinearity was analytically derived and investigated [17]. A total of 12 derived nonlinearity parameters were successfully used to detect cracks in metal components and turbine blades (Equation (2)). The excitation with two different frequencies was defined with $u^{\left(1\right)}=A_{1}sin\left[k_{f1}\left(x-ct\right)\right]+A_{2}cos\left[k_{f2}\left(x-ct\right)\right]$, where *A*_1_ and *A*_2_ are the amplitudes and *k_f_*_1_ and *k_f_*_2_ are the wavenumbers of the fundamental frequencies *f*_1_ and *f*_2_. $A_{f1+f2}$ are the amplitudes of the harmonic or modulated response frequencies:

(2) $$\begin{array}{c} \gamma_{f1}\propto \frac{A_{f1+f2}}{A_{1}{}^{3}+A_{1}A_{2}{}^{2}},\\ \gamma_{f2}\propto \frac{A_{f1+f2}}{A_{2}{}^{3}+A_{1}{}^{2}A_{2}},\\ \beta_{2f1}\propto \frac{A_{f1+f2}}{A_{1}{}^{2}},\\ \beta_{2f2}\propto \frac{A_{f1+f2}}{A_{2}{}^{2}},\\ \gamma_{3f1}\propto \frac{A_{f1+f2}}{A_{1}{}^{3}},\\ \gamma_{3f2}\propto \frac{A_{f1+f2}}{A_{2}{}^{3}},\\ \beta_{f2\pm f1}\propto \frac{A_{f1+f2}}{A_{1}A_{2}},\\ \gamma_{2f1\pm f2}\propto \frac{A_{f1+f2}}{A_{1}{}^{2}A_{2}},\\ \gamma_{2f2\pm f1}\propto \frac{A_{f1+f2}}{A_{1}A_{2}{}^{2}}. \end{array}$$

An overview of current research developments relating to nonlinear vibroacoustic modulation techniques is shown in [18]. Van Den Abeele et al. used two superimposed frequencies for excitation and examined the harmonics of both waves and their sideband frequencies to detect cracks [19]. They also developed a technique to investigate the influence of damaged materials on the amplitude-dependent resonance frequency shifts [20]. These methods are based on the nonlinear interactions of low-frequency and high-frequency waves. Straka et al. and Greenhall et al. worked on a nonlinear elastic wave modulation spectroscopy (NEWMS), where the effect of two superimposed waves was investigated, and low-frequency and high-frequency waves were combined to detect damages in components [21,22]. Using time-reversal-based imaging methods, an application for non-destructive material testing (NDT) with two multi-frequency signals was developed [23]. A technique was presented where two signals are correlated to estimate the Time-of-Flight (ToF) for determining distance information [24]. Novak et al. developed a method to extract system reactions for the second and third harmonic frequencies with a nonlinear convolutional signal analysis [25]. Pfleiderer et al. investigated the nonlinear response of subharmonics, ultra-subharmonics, and ultra-frequency pairs for detecting defects in components [26]. Abraham et al. used nonlinear multi-frequency ultrasound measurement to characterise grain size fluctuations and distributions in metal samples [27]. Gao et al. developed a method for estimating corrosion size, position, and depth in aircraft structures based on a local multi-frequency wavenumber estimation [28]. Malfense Fierro and Meo developed a phased array technique where three frequencies were used to identify cracks. The initiate signal *f*_2_ is sent out to affect the dynamics of the crack topology. The subsequent transmitted pump signals *f*_1_ and *f*_3_ generate a modulation that can be used for crack detection, and it was demonstrated that using multiple frequency excitation improves crack detection capabilities [29].

Deng investigated shear horizontal (SH) mode propagation on solid plates, where two shear waves propagate vertically and parallel in the plate and intersect. If the phase velocity of the SH mode is equal to the longitudinal velocity of the plate material, this leads to a cumulative effect of the second harmonic excitation frequency [30,31]. Pruell et al. showed that phase and group velocity matching can be used to generate acoustic nonlinearities to determine plasticity-driven fatigue damage [32,33]. Approaches were developed where correction factors were integrated into the solution of the wave equation for other wave types [34,35].

**Figure 1:**
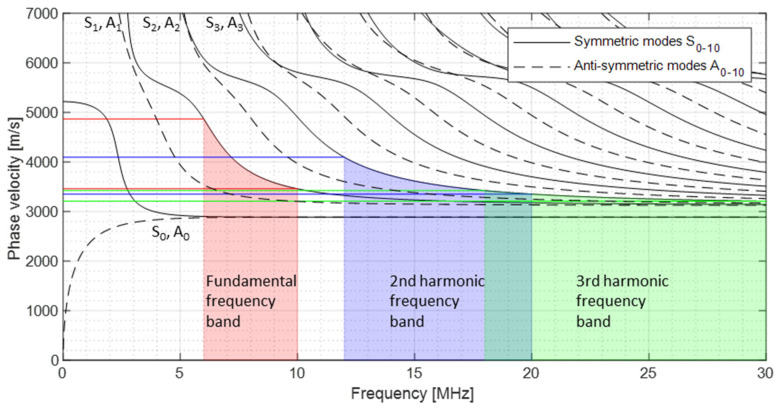
Dispersion curve—phase velocity.

To show possibly occurring Lamb waves, Figure 1 reveals the dispersion diagram of the phase velocity for the material Inconel 718, which is examined in Section 4. Due to comparable behaviour, the group velocity was not shown in this context. The fundamental frequencies from 6 MHz to 10 MHz were used in parallel and represented by the S_1_ mode according to [32]. The S_2_ mode demonstrates the range of the second harmonic frequency (12 MHz to 20 MHz) and S_3_ demonstrates the third harmonic frequency range (18 MHz to 30 MHz). It becomes clear that phase synchronisation with several excitation frequencies in this frequency range is not possible. The difference in phase velocities decreases only at higher frequencies, such as in the third harmonic frequency range. The integration of the modulated response frequencies would make synchronisation highly complex.

In this paper, the strength of the quasi-chaotic generation of harmonic and modulated response frequencies was clearly demonstrated, while the dispersion curves were not considered.

Different excitation concepts were compared to detect cracks in metal plates and on turbine blades. For this purpose, excitations with up to four frequencies were examined. In the case of quadruple excitation, a new concept of nonlinearity grouping was developed analytically. The fundamental frequencies were defined in such a way that several harmonics and sideband frequencies meet at certain frequencies to improve crack detection and classification.

## 2. Multiple Nonlinearity Parameter

Several studies demonstrated that damages in materials could be detected with nonlinear ultrasound techniques using two superimposed fundamental frequencies. The detection of defects was also significantly improved using three excitation frequencies, and therefore, the question is whether increasing this number brings further advantages.

### 2.1. Analytical Approach

To describe and evaluate the excitation with four frequencies, novel nonlinearity parameters up to the third-order of nonlinearity were derived analytically in this section.

**Figure 2:**
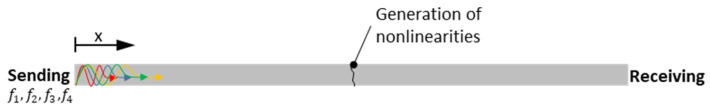
Model—multi-frequency excitation.

Figure 2 demonstrates this excitation with the one-dimensional model. In the area of the defect, nonlinearities are generated, and these signals are received at the other end.

The one-dimensional wave equation was solved with four excitation frequencies. All harmonic frequencies and modulated response frequencies up to the third-order were derived as well as the associated nonlinearity parameters, which include the amplitudes of the fundamental frequencies and the harmonic and sideband frequencies as variables.

In the presence of nonlinearity, the stress σ is defined as:

(3) $$\sigma =E\varepsilon +\frac{E\beta }{2}\varepsilon^{2}+\frac{E\gamma }{6}\varepsilon^{3},$$

where *E* is the Young’s modulus, ε is the strain, β is the second-order nonlinearity parameter, and γ is the third-order nonlinearity parameter.

Equation (4) is the one-dimensional wave equation, with the assumption that longitudinal waves propagate in a thin circular rod and the attenuation is neglected:

(4) $$\rho \frac{\partial^{2}u}{\partial t^{2}}=\frac{\partial \sigma }{\partial x}$$

where ρ is the mass density and *u* represents the displacement. The wave speed is defined as $c=\sqrt{\frac{E}{\rho }}$ and the strain as $\varepsilon =\frac{\partial u}{\partial x}$. This is substituted into Equations (3) and (4) and leads to:

(5) $$\underset{\mathrm{Linear}\;\mathrm{part}}{\underbrace{\frac{\partial^{2}u}{\partial t^{2}}-c^{2}\frac{\partial^{2}u}{\partial x^{2}}}}=\underset{\mathrm{Nonlinear}\;\mathrm{part}}{\underbrace{\underset{2nd\;\mathrm{order}}{\underbrace{c^{2}\beta \frac{\partial u}{\partial x}\frac{\partial^{2}u}{\partial x^{2}}}}+\underset{3rd\;\mathrm{order}}{\underbrace{\frac{c^{2}\gamma }{2}{\left(\frac{\partial u}{\partial x}\right)}^{2}\frac{\partial^{2}u}{\partial x^{2}}}}}}.$$

Equation (5) is solved using the perturbation method; therefore, Equation (6) is used to find solutions for $u^{\left(2\right)}$ with the second-order parameter *β* and $u^{\left(3\right)}$ with the third-order parameter *γ*:

(6) $$u=u^{\left(1\right)}+u^{\left(2\right)}+u^{\left(3\right)}$$

According to Fourier, the assumption for $u^{\left(1\right)}$ for four excitation frequencies is:

(7) $$\begin{array}{c} u^{\left(1\right)}=A_{1}sin\left[k_{f1}\left(x-ct\right)\right]+A_{2}cos\left[k_{f2}\left(x-ct\right)\right]\\ +A_{3}sin\left[k_{f3}\left(x-ct\right)\right]+A_{4}cos\left[k_{f4}\left(x-ct\right)\right], \end{array}$$

where *A*_1,2,3,4_ are the amplitudes and *k*_1,2,3,4_ are the wavenumbers of the four fundamental frequencies. The detailed derivations are documented in Appendix A.

With an assumed constant wave propagation distance and wavenumber, the general expressions in Table 1 can be derived. The displacement *u* (Equations (A2) and (A5)) is interpreted as the accumulated amplitude of the harmonic frequency: *u*(*x*) = *A*_*f*1+*f*2+*f*3+*f*4_.

The parameters *β* or *γ* were used depending on the degree of nonlinearity. In the following, *δ* is used overriding and independent of the degree of nonlinearity for all nonlinearity parameters. A comparison of the various nonlinearity parameters due to multi-frequency excitation is shown in Appendix B.

### 2.2. Group Parameters

The choice of excitation frequencies is crucial to improving damage detection. The following criteria were defined for selecting the highest (*f*_4_) and lowest frequency (*f*_1_) in the multi-frequency analysis:

1. No frequency overlap: $f_{4}\neq \frac{f_{1}}{2}$.
2. No frequencies with theoretical zero values and negative values: $f_{4}>\frac{f_{1}}{2}$.
3. Sensor bandwidth.

With the defined criteria, the excitation frequencies *f*_1_ = 6.8 MHz, *f*_2_ = 7.3 MHz, *f*_3_ = 10 MHz, and *f*_4_ = 12 MHz result in 64 individual harmonic and sideband frequencies. If the excitation frequency combination is chosen systematically (*f*_1_ = 6 MHz, *f*_2_ = 7.3 MHz, *f*_3_ = 8.7 MHz, and *f*_4_ = 10 MHz), only 30 harmonic and sideband frequencies result. The signal energies of the various harmonic and sidebands add up at specific frequencies; therefore, the group parameters form a stronger indicator for the presence of nonlinearities. The nonlinearity parameters shown in Table 1 describe individual values for each frequency. When different fundamental frequencies, harmonic frequencies, and sidebands coincide on a frequency, these parameters are no longer valid. The nonlinearity information of one frequency must be summarised on one nonlinearity parameter. For this purpose, the solutions from *u*^(2)^ (Equation (A2)) and *u*^(3)^ (Equation (A5)) were re-examined for the grouped nonlinearity parameters. If, for example, the sideband frequencies *f*_3_ − *f*_1_ and *f*_4_ − *f*_2_ coincide at the frequency 2.6 MHz, it can be assumed that the individually calculated nonlinearity parameters, *δ*, are identical at this point. The total displacement, according to Equations (A2) and (A5), is defined as $u=u_{f_{3}-f_{1}}+u_{f_{4}-f_{2}}$, which is interpreted as the accumulated amplitude of the sideband frequency (2.6 MHz). Therefore, equations were solved for the new combined nonlinearity parameters. Thirty new parameters were derived for this work, called group parameter *δ_G_*. Table 2, Table 3, Table 4 and Table 5 illustrate the groups with corresponding frequencies, the harmonic and sideband designation, and the new group nonlinearity parameters *δ_G_*.

The derivation of this parameter is illustrated by Equation (8). For group 1, where only one harmonic or sideband per frequency were used, the parameters result from Equations (A2) and (A5). In group 2, two harmonics and sidebands always meet one frequency, which is described by the generalised shift of group 2 with *u*_*G*2_. The amplitudes *A*_1_ and *A*_2_ reveal the summary of the solutions from Equations (A2) and (A5) which should be solved. The placeholder $\Gamma_{f/h/s}$ represents the combination of fundamental, harmonic, and sideband frequencies:

(8) $$\begin{array}{c} u_{G2}=A_{1}\begin{array}{c} sin\\ cos \end{array}\left[\left(\Gamma_{f/h/s_{1}}\right)\left(x-ct\right)\right]x+A_{2}\begin{array}{c} sin\\ cos \end{array}\left[\left(\Gamma_{f/h/s_{2}}\right)\left(x-ct\right)\right]x,\;\\ u_{G3}=A_{1}\begin{array}{c} sin\\ cos \end{array}\left[\left(\Gamma_{f/h/s_{1}}\right)\left(x-ct\right)\right]x\\ +A_{2}\begin{array}{c} sin\\ cos \end{array}\left[\left(\Gamma_{f/h/s_{2}}\right)\left(x-ct\right)\right]x+A_{3}\begin{array}{c} sin\\ cos \end{array}\left[\left(\Gamma_{f/h/s_{3}}\right)\left(x-ct\right)\right]x,\\ u_{G4}=A_{1}\begin{array}{c} sin\\ cos \end{array}\left[\left(\Gamma_{f/h/s_{1}}\right)\left(x-ct\right)\right]x+A_{2}\begin{array}{c} sin\\ cos \end{array}\left[\left(\Gamma_{f/h/s_{2}}\right)\left(x-ct\right)\right]x\\ +A_{3}\begin{array}{c} sin\\ cos \end{array}\left[\left(\Gamma_{f/h/s_{3}}\right)\left(x-ct\right)\right]x+A_{4}\begin{array}{c} sin\\ cos \end{array}\left[\left(\Gamma_{f/h/s_{4}}\right)\left(x-ct\right)\right]x. \end{array}$$

This procedure was also used for the further displacement terms $u_{G3}$ and $u_{G4}$.

**Figure 3:**
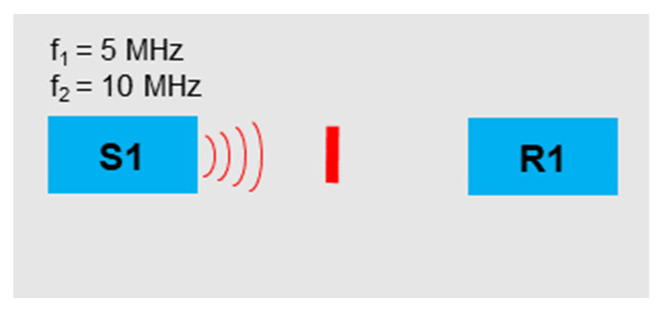
Sensor arrangement—evidence signal grouping.

If several harmonics or sidebands meet on one frequency, it was expected that the signal energy increases at this point. This assumption was proved experimentally, and the groupings were conducted with two frequencies for comparison. In this case, the previously defined criteria for frequency combination selection do not apply. The frequencies *f*_1_ = 5 MHz and *f*_2_ = 10 MHz are selected for the experiments (Figure 3).

Using the pulse generator Rigol DG1022Z, the sinus signals were sent to the sensor Olympus A5013. The same sensor was used on the receiving side. Table 6 illustrates the fundamentals, harmonics, and sidebands with the corresponding frequencies and groupings. With this frequency combination, there are several harmonics and sidebands at the frequencies 5 MHz, 10 MHz, 15 MHz, and 20 MHz.

**Figure 4:**
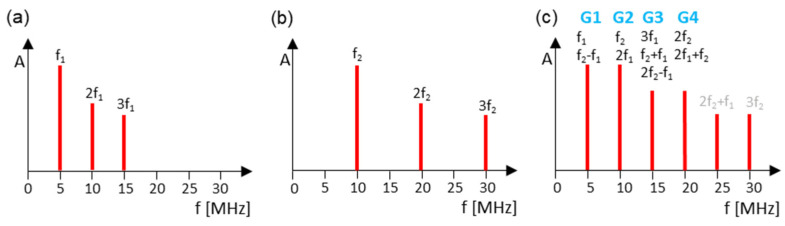
Grouping spectra: (**a**) *f*_1_ = 5 MHz; (**b**) *f*_2_ = 10 MHz; (**c**) *f*_1_ = 5 MHz and *f*_2_ = 10 MHz.

The first measurement was separately executed with one frequency, *f*_1_ and *f*_2_. In the frequency spectrum, the amplitudes were measured at positions *f*_1/2_, 2*f*_1/2_, and 3*f*_1/2_ (Figure 4a,b). The same experiments were repeated using two superimposing frequencies (*f*_1_ and *f*_2_) transmitted in parallel (Figure 4c). For this purpose, 100 signals were sent out, an average frequency spectrum was formed, and the corresponding amplitudes were evaluated.

**Figure 5:**
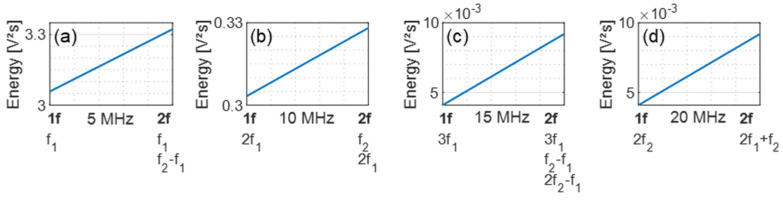
Energy comparison: (**a**) Group 1 (5 MHz); (**b**) Group 2 (10 MHz); (**c**) Group 3 (15 MHz); (**d**) Group 4 (20 MHz).

The signal energies were calculated from the measured amplitudes and shown as a comparison in Figure 5. The signal energy resulting from the excitation with one frequency is on the left side of the graphs (1f), while the energy from the two-frequency excitation is demonstrated on the right (2f). It is evident that more energy was transmitted in the multi-harmonic and sideband grouped signals (2f).

### 2.3. Evaluation Parameter

The aim of this work is to demonstrate a practicable application of the multi-frequency excitation and compare the results with one, two, and four excitation frequencies.

In other research, the total sums of the fundamental frequencies and those of the harmonic and sideband frequencies were compared for evaluation (Equation (9)) [17,36]:

(9) $$E=\int_{-\infty }^{\infty }{\left|\hat{x}(f_{n})\right|}^{2}df_{n}\propto \sum \left|A_{F}\right|+\sum \left|A_{H}\right|\propto \sum \left|E_{F}\right|+\sum \left|E_{H}\right|,$$

where *E* is the energy, $\hat{x}$ is the signal, *A_F_* are the fundamental amplitudes, *A_H_* are the harmonic amplitudes, $E_{F}$ is the signal energy of the fundamental frequencies, and $E_{H}$ is the energy of the harmonic signal. This approach may not be applicable with the four-frequency excitation, however, because the grouping of the fundamental frequencies as well as the harmonic and sideband frequencies may overlap. Another possibility of evaluation with the same disadvantages is to use the acoustic moment concept, which is the integral of the power spectral density (PSD) function of a signal [37,38].

In addition to the nonlinearity parameters, the comparison value *N_index_* was introduced for this purpose. This is a summation of the parameter values δ (Equation (10)):

(10) $$N_{index}^{\left(1f,2f,2f-swept,\;4f\right)}=\overset{m}{\underset{n=1}{\sum }}\left|\delta_{n}\right|,$$

where *m* is the total number of nonlinearity parameters of the individual measurements.

## 3. Proof of the Use of the One-Dimensional Wave Equation

**Figure 6:**
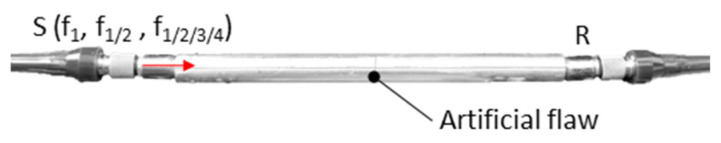
Experimental setup—longitudinal waves.

The derived nonlinearity parameters are based on one-dimensional wave propagation, and it was examined as to whether this model can also be applied to multi-dimensional applications. A round bar made of the material 1.4571 with a diameter of 5 mm and length of 100 mm was used to simulate a one-dimensional model. To generate artificial contact nonlinearities, the sample was cut in the middle and glued back together. At an area of 1 mm wide, no glue was used so that the two halves had metallic contact. For the measurements, the Olympus sensor A109S was used to send the longitudinal waves into the sample via the front side and to receive them on the other side (Figure 6).

One frequency (6 MHz), two frequencies (6 and 10 MHz), and 4 frequencies (6, 7.3, 8.7, and 10 MHz) were sent according to the group derivation from Section 2.2. The signals were generated with Rigol DG1022Z pulse generators over two channels each and an input voltage of 150 V. The output signal was amplified with the Phoenix ISL 40 dB amplifier.

**Figure 7:**
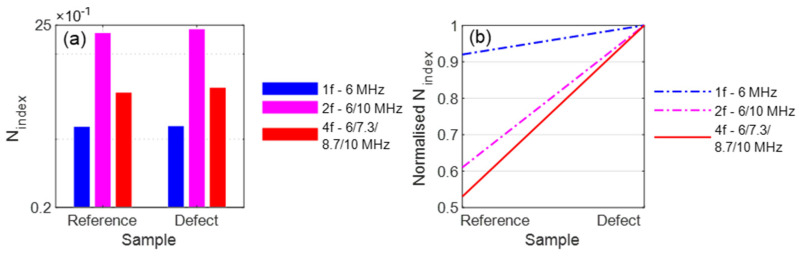
Evaluation: (**a**) *N_index_*; (**b**) Normalised *N_index_*.

These tests were also conducted with a reference sample without damage for comparison. The nonlinearity parameters were calculated from these measurements, and the determined *N_index_* values are shown in Figure 7a. To clarify the comparison of the excitation methods, the normalised view was also selected (Figure 7b). It becomes clear that the difference with the 4f excitation is stronger than with the 1f excitation.

## 4. Experimental Investigation—Plate Samples

In this section, the analytical derivations were proven experimentally and compared with the different excitation concepts.

### 4.1. Experimental Setup

**Figure 8:**
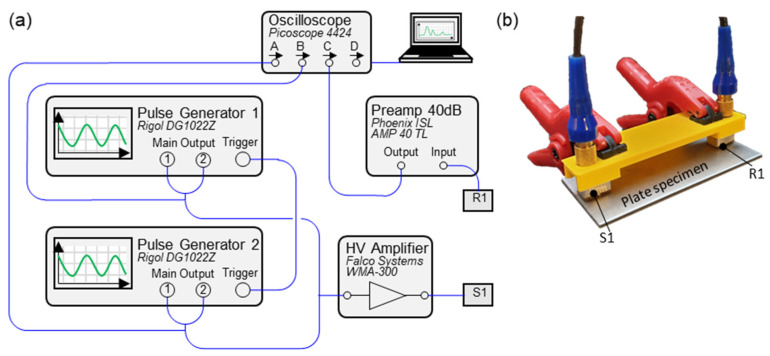
Experimental setup: (**a**) Scheme; (**b**) Sensor arrangement.

Identical test setups were installed for all experiments (Figure 8a), where two Rigol DG1022Z pulse generators were used for signal generation. Each device had two output channels to send the single or superimposed frequencies to the sending sensor. For synchronisation, both devices were connected to each other via the trigger inputs and outputs. The combined signals were amplified with the amplifier Falco WMA-300. The S1 sensor was the Olympus A5013. The same sensor was used to receive the signal (R1), which was amplified with the Phoenix ISL 40 dB amplifier and sent to the Picoscope 4424 oscilloscope. The post-processing was conducted from this position.

Figure 8b reveals the experimental setup. The sensors were held in an additively manufactured holder and always ensured precise alignment. Two clamps were used for a certain contact pressure, and standard contact gel was applied under the sensors for better contact.

**Figure 9:**
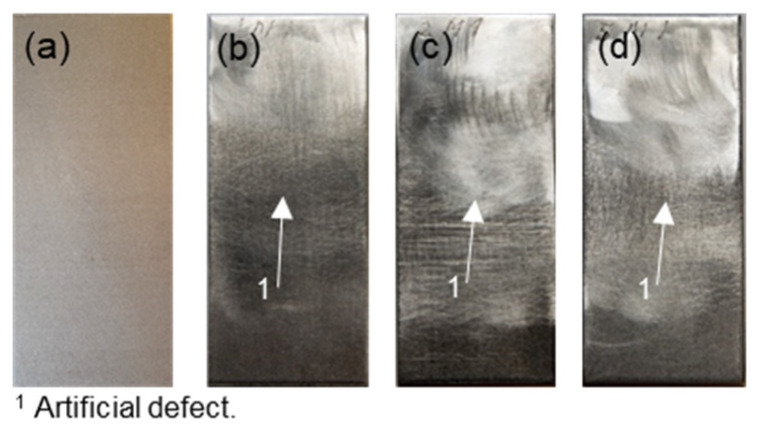
Specimen: (**a**) X1—reference sample; (**b**) 1M—failure size: 1 mm; (**c**) 2M—failure size: 2 mm; (**d**) 5M—failure size: 5 mm.

The samples used were Inconel 718 plates with the dimensions 2 mm × 70 mm × 30 mm, which consisted of two plates connected using a special micro laser welding process. Samples with a defect width of 5, 2, and 1 mm were used and arranged centrally (Figure 9). Due to these artificial defects, contact nonlinearities were generated and evaluated, and reference samples without defects were available for comparison. Six variants of each sample were examined, and the arithmetic mean values were processed.

### 4.2. One-Frequency Excitation

**Figure 10:**
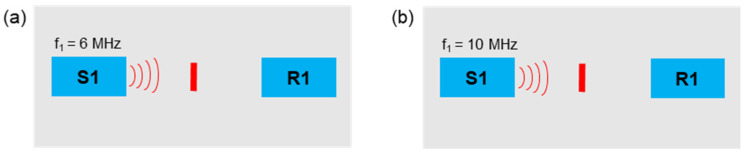
Excitation frequencies: (**a**) *f*_1_ = 6 MHz; (**b**) *f*_1_ = 10 MHz.

In this section, the excitation via single frequencies was examined. A 6 MHz frequency signal and a 10 MHz signal were separately sent to the samples (Figure 10).

**Figure 11:**
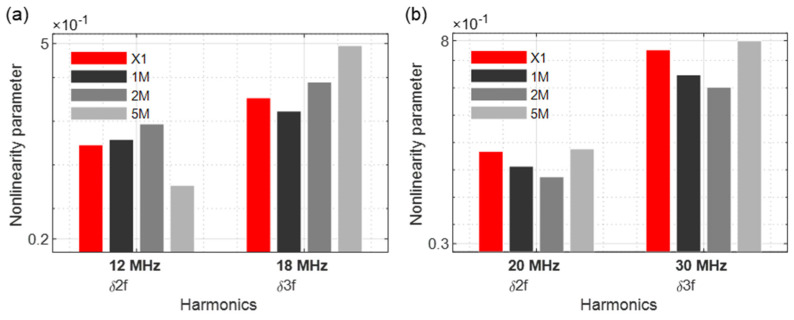
Nonlinearity parameter: (**a**) *f*_1_ = 6 MHz; (**b**) *f*_1_ = 10 MHz.

The second and third harmonic frequencies were considered accordingly. When 6 MHz is excited, the nonlinearity parameters of samples 1M and 2M increase significantly (Figure 11a). A form of saturation was observed as the values drop in the 5M sample. With the nonlinearity parameters of the third harmonic frequency, the value for the 1M sample decreases and then continuously increases for the other samples (2M and 5M). If the samples are sonicated with 10 MHz, a downward trend for both the second and third harmonic frequencies was given. Only the 5M sample demonstrated a significant increase in nonlinearities compared to the reference sample (Figure 11b).

### 4.3. Two-Frequency Excitation

#### 4.3.1. Constant Frequencies

**Figure 12:**
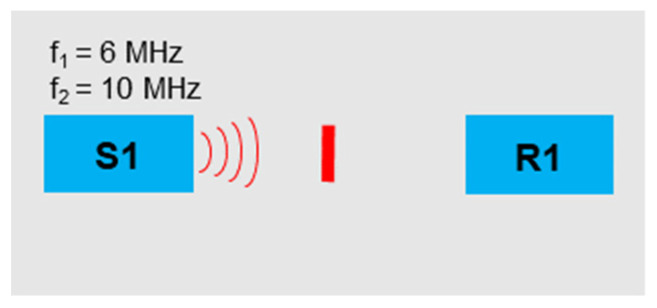
Excitation frequencies: *f*_1_ = 6 MHz, *f*_2_ = 10 MHz.

If two frequencies emitted in parallel are used, the evaluation becomes more complex since a total of 12 harmonic and sideband frequencies are formed which can be evaluated. This technique was successfully used and proven in [17]. A 6 MHz and 10 MHz signal were generated, superimposed, and sent via sensor S1 (Figure 12). If the waves overlap, harmonic frequencies and more complex modulated response frequencies were generated, which are evaluated in the following. To compensate the overlapping effects of alternating compressive and tensile stresses on the various sine waves, 100 signals were transmitted per experiment and the mean values of the amplitudes were evaluated.

**Figure 13:**
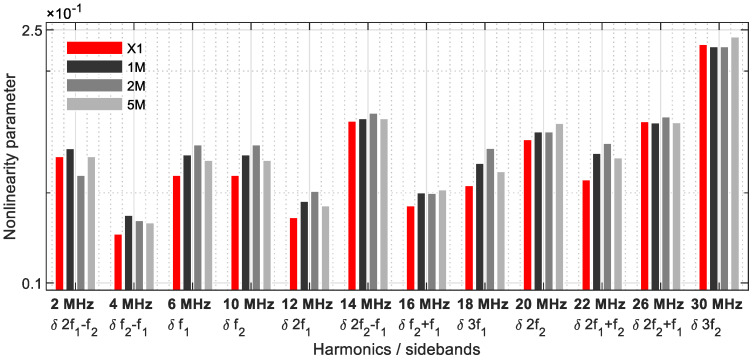
Nonlinearity parameter.

Figure 13 reveals the evaluation of the nonlinearity parameters, and all demonstrate a change compared to the reference sample. The parameter value *δ*2*f*_2_ showed a clear pattern depending on the crack size. This phenomenon was moreover observed in [17,36], where the largest nonlinearities were not generated for the largest crack. These are strongly dependent on the fundamental frequencies and illustrate significantly more influence with the 1M and 2M samples.

#### 4.3.2. Linear Increasing Frequency

**Figure 14:**
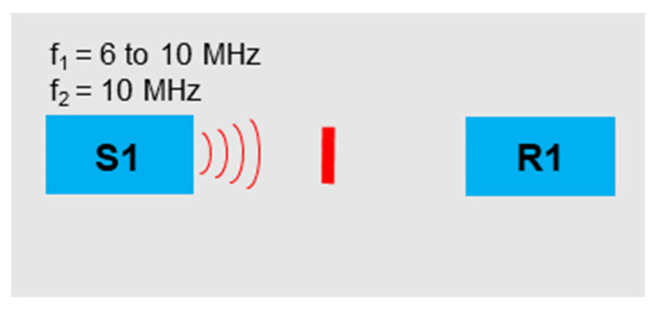
Excitation frequencies: *f*_1_ = 6 to 10 MHz, *f*_2_ = 10 MHz.

To investigate as many frequency combinations as possible, the concept of linearly increasing frequencies was also explored. A constant frequency of 10 MHz was transmitted via the sensor S1, and a 6 MHz signal was sent in parallel, which increases linearly to 10 MHz over a period of 60 s. The signals were received by sensor R1 (Figure 14), and 60 measurements per second were recorded.

**Figure 15:**
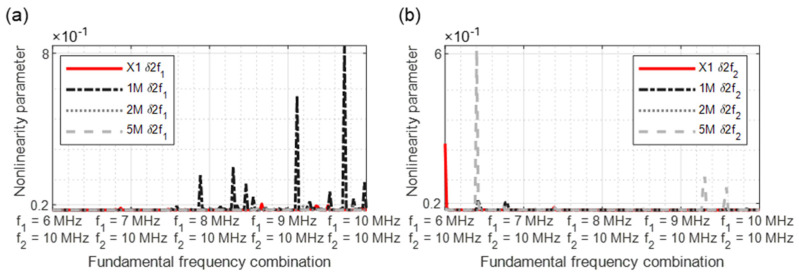
Nonlinearity parameter: (**a**) *δ*2*f*_1_; (**b**) *δ*2*f*_2_.

**Figure 16:**
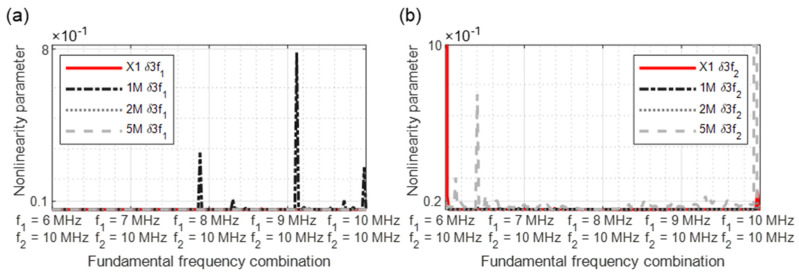
Nonlinearity parameter: (**a**) *δ*3*f*_1_; (**b**) *δ*3*f*_2_.

**Figure 17:**
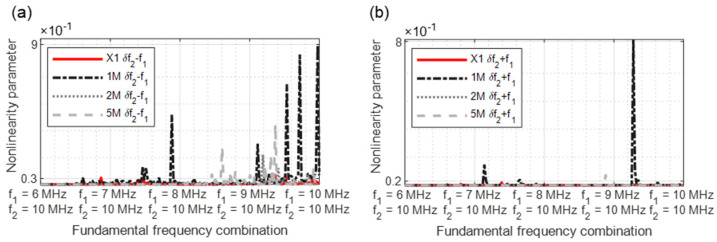
Nonlinearity parameter: (**a**) *δf*_2_ − *f*_1_; (**b**) *δf*_2_ + *f*_1_.

**Figure 18:**
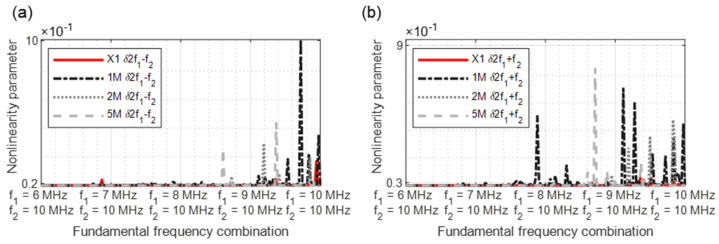
Nonlinearity parameter: (**a**) *δ*2*f*_1_ − *f*_2_; (**b**) *δ*2*f*_1_ + *f*_2_.

**Figure 19:**
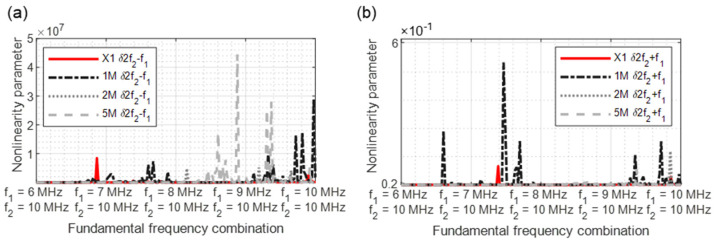
Nonlinearity parameter: (**a**) *δ*2*f*_2_ − *f*_1_; (**b**) *δ*2*f*_2_ + *f*_1_.

In Figure 15, Figure 16, Figure 17, Figure 18 and Figure 19, the nonlinearity parameters are shown for the individual harmonics and sidebands.

In Figure 15a, the x-axis illustrates the actual increasing frequency combination starting with 6/10 MHz up to 10/10 MHz. At the excitation frequencies of 8.4/10 MHz, the values of the 5M sample increase drastically. The values of the 1M sample are consistently higher than those of the 2M sample, while the reference values are always lower, except for a peak at 7.4/10 MHz. With parameter *δ*2*f*_2_ (Figure 15b), there were increased nonlinearities in the 5M sample at the frequency combinations 6.4/10 MHz, 9.25/10 MHz, and 9.6/10 MHz; therefore, the 1M sample reveals smaller peaks at 6.4/10 MHz and 7.8/10 MHz.

The third harmonic frequency of the sample 1M demonstrates several significant points at *δ*3*f*1 (Figure 16a). With *δ*3*f*2, increased material nonlinearities were detected in the reference sample at the start of the measurement. In the further course, several peaks of the 5M sample arise again (Figure 16b).

In Figure 17a, the strength of the dual frequency excitation with a modulated response parameter becomes clear. A comparison demonstrates that the *δf*_2_ − *f*_1_ values for the 1M sample as well as for the 2M and 5M samples reveal significant increases in nonlinearity. For the parameter *δf*_2_ + *f*_1_, all samples reveal peaks from different frequency excitation combinations (Figure 17b).

The parameter *δ*2*f*_1_ − *f*_2_ (Figure 18a) demonstrates a clear crack-predicting behaviour. All damaged samples reveal nonlinear features. The previously shown nonlinearity behaviour is continued in Figure 18b, with clear nonlinear signature particularly in the frequency range 9/10 MHz to 10/10 MHz.

In Figure 19a, the sample 1M stands out again due to increased parameter values. In the higher frequency combination range, the damages of the samples 2M and 5M could be identified. Of all parameters, 2*f*_2_ + *f*_1_ (Figure 19b) best demonstrates the nonlinearity increases as a function of the crack size. Although higher values were required from the frequency combination 7/10 MHz, the clearest results are from 8.6/10 MHz to 10/10 MHz.

### 4.4. Four-Frequency Excitation

**Figure 20:**
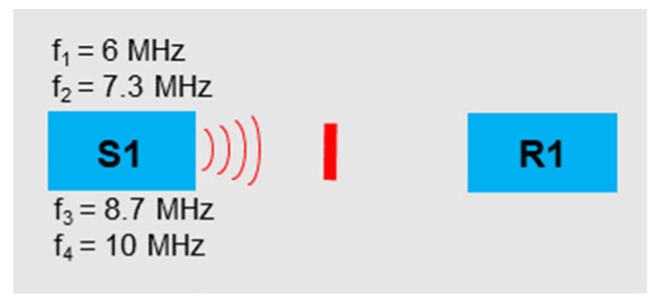
Excitation frequencies: *f*_1_ = 6 MHz, *f*_2_ = 7.3 MHz, *f*_3_ = 8.7 MHz, *f*_4_ = 10 MHz.

Four frequencies were sent into the sample with sensor S1 (Figure 20). The frequency selection should relate to the concept of frequency grouping (Section 2.2).

**Figure 21:**
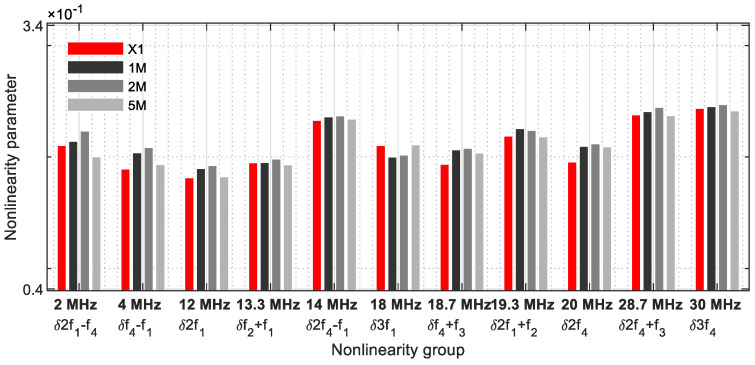
Nonlinearity parameter—*δ*_*G*1_.

**Figure 22:**
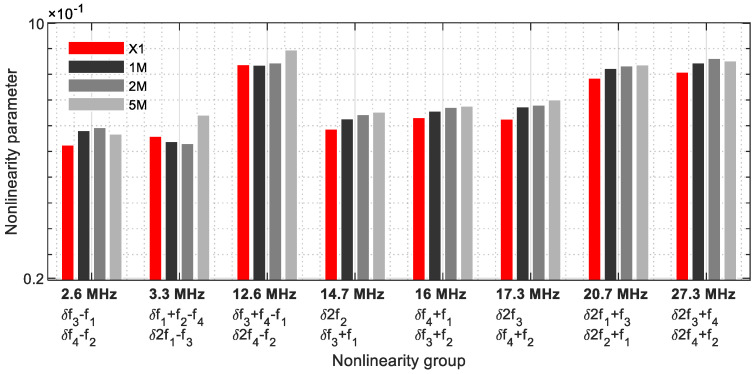
Nonlinearity parameter—*δ*_*G*2_.

**Figure 23:**
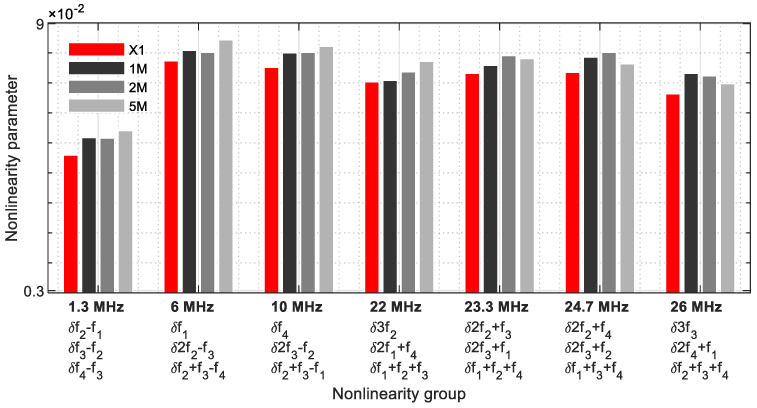
Nonlinearity parameter—*δ*_*G*3_.

**Figure 24:**
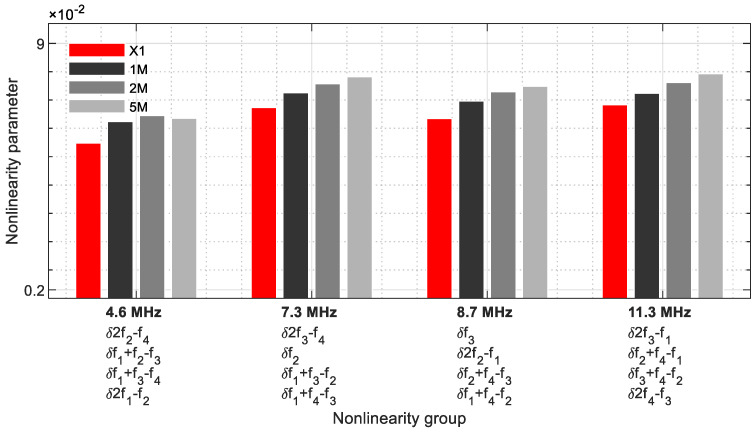
Nonlinearity parameter—*δ*_*G*4_.

In Figure 21, Figure 22, Figure 23 and Figure 24, the nonlinearity parameters were shown separately with the different groupings. Figure 21 demonstrates all nonlinearity parameters for one frequency (Table 2). The parameter values of the damaged samples usually differ significantly from those of the reference sample. With the 5M sample, insufficient nonlinearities are generated to observe a clear change to the reference sample. For the parameters *δ*2*f*_1_, *δf*_2_ + *f*_1_, *δ*2*f*_4_ − *f*_1_, *δ*3*f*_1_, *δ*2*f*_1_ + *f*_2_, *δ*2*f*_4_ + *f*_3_, and *δ*3*f*_4_, the values of the reference sample and the damaged 5M sample are on the same level. With this nonlinearity group, frequencies with a large bandwidth from 2 MHz to 30 MHz were considered.

In the nonlinearity grouping with 2 frequencies (Table 3), eight parameters were evaluated (Figure 22), with a clear change to the reference sample. At 12.6 MHz (δ*f*_3_ + *f*_4_ − *f*_1_/δ2*f*_4_ − *f*_2_), 14.7 MHz (δ2*f*_2_/δ*f*_3_ + *f*_1_), 16 MHz (δ*f*_4_ + *f*_1_/δ*f*_3_ + *f*_2_), 17.3 MHz (2*f*_3_/δ*f*_4_ + *f*_2_) and 20.7 MHz (δ2*f*_1_ + *f*_3_/δ2*f*_2_ + *f*_1_), there is an increase in the nonlinearity parameters as a function of the crack size. The bandwidth ranges from 2.6 MHz to 27.3 MHz.

Figure 23 demonstrates the frequencies where three nonlinearity parameters meet (Table 4). This group consists of seven frequencies covering a bandwidth from 1.3 MHz to 26 MHz. These results revealed the strength of the multiple harmonic and modulated response frequencies on one frequency. They reflect the size of the cracks in the samples, with exception of the higher frequencies (23.3 MHz, 24.7 MHz, and 26 MHz). Contrary to group 1, an increase in the parameter values of the 5M sample is given; however, the values remain below the 1M and 2M samples.

The group with four nonlinearity parameters per frequency is shown Figure 24 (Table 5), which was revealed in a narrower frequency range from 4.6 MHz to 11.3 MHz. This illustrated that the energy of four fundamental, harmonic, and sideband frequencies are combined at one frequency, which is why this group has the greatest influence in the evaluation. At 7.3 MHz and 8.7 MHz, two fundamental frequencies are included. At 4.6 MHz, the values of the 5M sample decrease. The other frequencies reveal the nonlinearity parameters as a function of the crack size.

### 4.5. Results and Discussion

**Figure 25:**
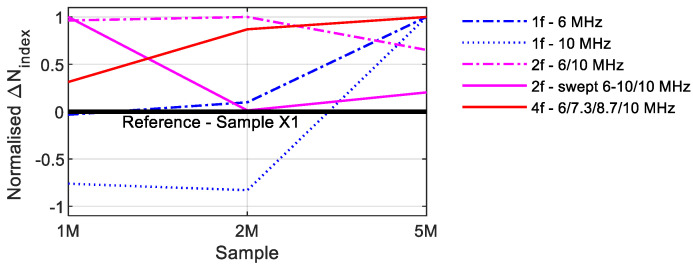
Comparison *N_index_* values.

The various excitation techniques are compared in this section. Therefore, Figure 25 illustrates all *N_index_* values. Since the reference sample X1 represents the basis of comparison (*N_index_* = 0), only the difference to these values were shown. If the values are higher than zero, the nonlinearities increase; if they are smaller, the nonlinearities decrease. The values drop sharply with single-frequency excitation at 10 MHz. Only the values of the 5M sample are in the positive range. The 6 MHz excitation provided better values, even if the values of the 1M sample are minimally below the value of the reference sample. The constant excitation with two frequencies reveals a clear increase in the values, which, however, decrease in the still-positive range for the 5M sample. The arithmetic mean of all measurement data was used to evaluate the swept values. The *N_index_* of the 2M sample is on the same level as the reference sample. In this graphic, it was only possible to deliver a course as a function of the crack size with the quadruple-excitation.

## 5. Experimental Investigation—Turbine Blades

In the previous section, the various excitation philosophies were tested on metal plates with artificial defects. In this section, turbine blades with cracks on the trailing edges were examined using the different excitation concepts.

### 5.1. Experimental Setup

**Figure 26:**
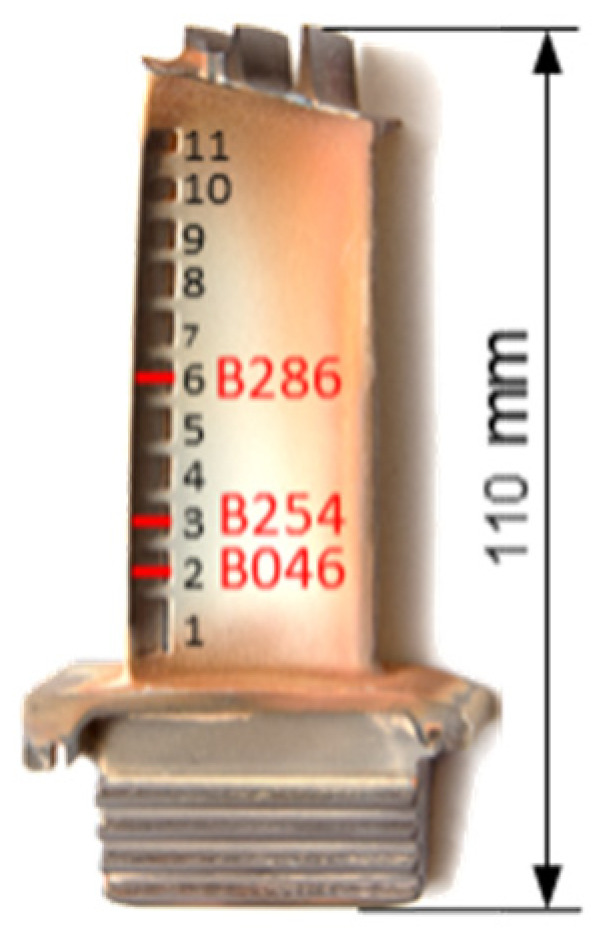
Damaged turbine blades.

Four Inconel 628 turbine blades from an industrial gas turbine were used for this study. Three blades had cracks on the trailing edges on different positions, while one blade was unused and undamaged and serves as a reference for comparison. These blades are cooled internally, with the cooling air directed into the blade via the blade root and exiting via various outlet slots at the trailing edge following the serpentine cooling. Figure 26 illustrates the blades used with the crack positions and Table 7 illustrates the corresponding crack lengths.

**Figure 27:**
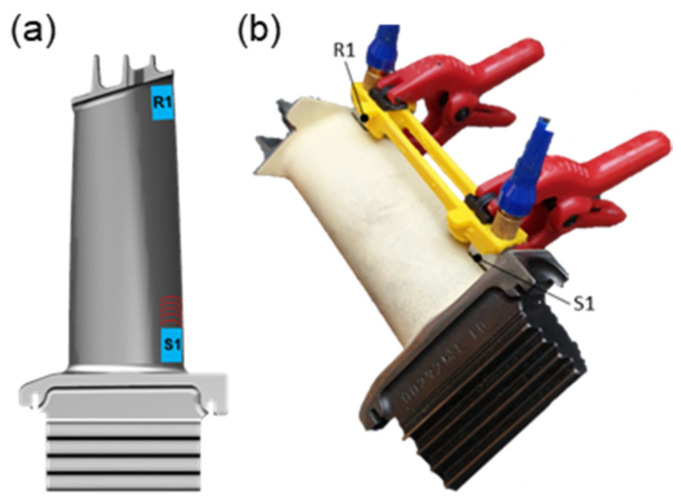
Measurements of turbine blades: (**a**) Sensor orientation; (**b**) Experimental setup.

The experimental setup was implemented as described in Section 4. The emitting sensor S1 was positioned in the area of the inner shroud on a smooth area on the blade airfoil (Figure 27a). The receiving sensor R1 was located opposite, near the outer shroud. Using an additively manufactured sensor holder, the sensors are held in the same position during all tests, and an identical measuring distance was ensured (Figure 27b).

### 5.2. Measurements with Results and Discussion

**Figure 28:**
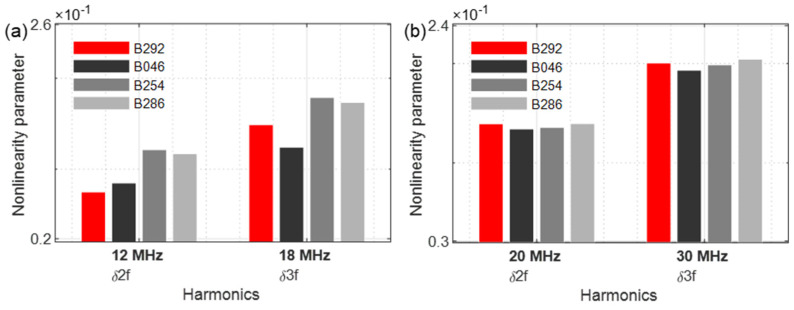
Nonlinearity parameter: (**a**) *f*_1_ = 6 MHz; (**b**) *f*_1_ = 10 MHz.

Figure 28 revealed the excitation with a frequency of 6 MHz (Figure 28a) and 10 MHz (Figure 28b). When excited with 6 MHz, the second harmonic parameter *δf*_2_ demonstrates a clear increase in nonlinearities; the 10 MHz excitation, however, hardly allows conclusions to be drawn about the damage.

**Figure 29:**
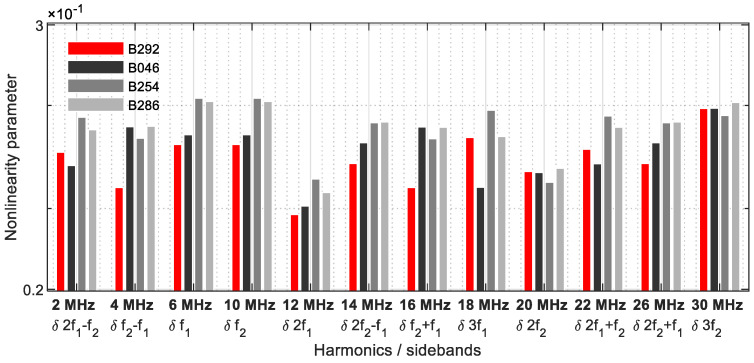
Nonlinearity parameter.

The dual frequency excitation with 6 MHz and 10 MHz clearly reveals increases in nonlinearity (Figure 29).

**Figure 30:**
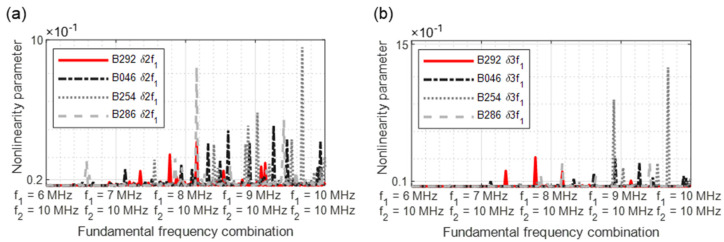
Nonlinearity parameter: (**a**) *δ*2*f*_1_; (**b**) *δ*3*f*_1_.

**Figure 31:**
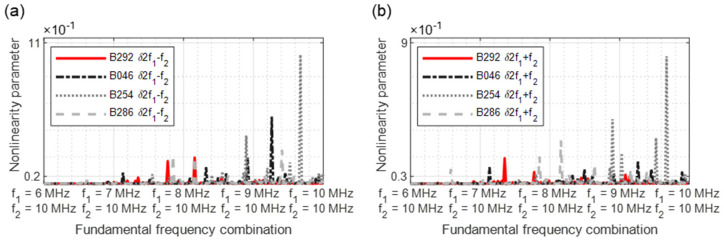
Nonlinearity parameter: (**a**) *δ*2*f*_1_ − *f*_2_; (**b**) *δ*2*f*_1_ + *f*_2_.

In Figure 30 and Figure 31, the most relevant results are demonstrated with swept signals. It is noticeable that the reference blades (B292) also have a higher degree of nonlinearity peaks (Figure 30a). Using an excitation frequency combination starting with 8.2/10 MHz, both the reference blade B292 and blade B286 show harmonics. Further significant increases in the nonlinearity parameters were measured for all damaged blades. As with the metal plate samples, the excitation frequency combinations from 8/10 MHz lead to considerable increases in the nonlinearity parameters. Blade B254 achieved the highest values for both *δ*2*f*_1_ and parameter *δ*3*f*_1_ (Figure 30b).

The results of two modulated response parameters (δ2*f*_2_ ± *f*_1_) are illustrated in Figure 31. The blade B254 reveals the highest nonlinear values. The crack can also be diagnosed for blades B046 and B286 by the increased nonlinearity values.

**Figure 32:**
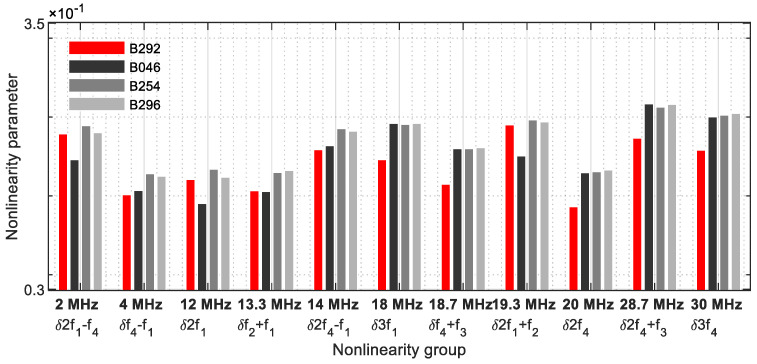
Nonlinearity parameter—*δ*_*G*1_.

When evaluating the excitation with four frequencies, the procedure described in Section 2.2 was followed. The grouping of the nonlinearity parameters was used for crack detection. Figure 32 illustrates group 1, with one nonlinearity parameter per frequency. The parameters *δf*_4_ − *f*_1_, *δ*2*f*_4_ − *f*_1_, *δ*3*f*_1_, *δf*_4_ + *f*_3_, *δ*2*f*_4_, *δ*2*f*_4_ + *f*_3_, and *δ*3*f*_4_ reveal a clear increase in the nonlinearity parameters and allow a positive crack prognosis.

**Figure 33:**
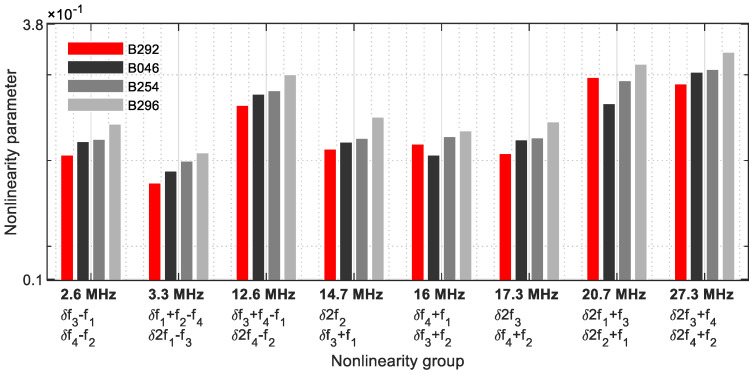
Nonlinearity parameter—*δ*_*G*2_.

If two parameters decrease on one frequency (Figure 33), the results are strong, showing that detecting cracks was possible with this group evaluation alone.

**Figure 34:**
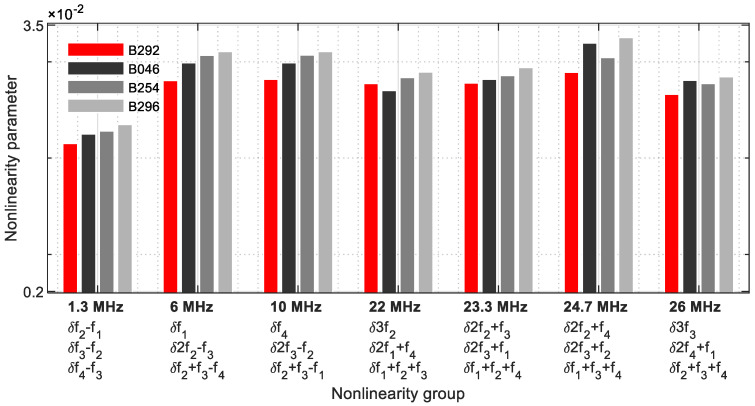
Nonlinearity parameter—*δ*_*G*3_.

This behaviour continues and is also shown in the nonlinearity group 3 (Figure 34). Only *δ*3*f*_2_ failed to clearly detect the crack on the blade B046.

**Figure 35:**
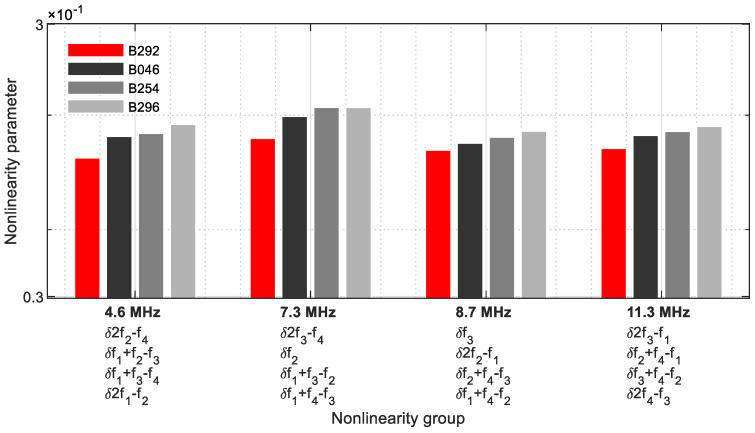
Nonlinearity parameter—*δ*_*G*4_.

As in Section 4, the differences between the various parameter values have become smaller (Figure 35). Because the signal energies of four fundamental, harmonic, and modulated response parameters each coincide on one frequency, this group approach provides the strongest informative value.

**Figure 36:**
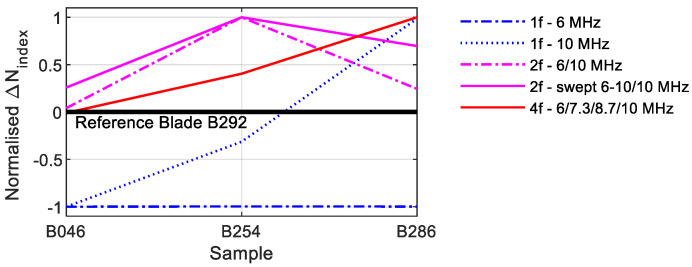
Comparison *N_index_* values.

In Figure 36, the normalised *N_index_* values for the different processes are used for evaluation again. If only one frequency is used for excitation, a crack forecast with these values is hardly possible; if two frequencies are used, both constant excitation and excitation by means of swept signals reveal a comparable, increasing behaviour; with four frequency excitations, these values increase continuously.

In most of the measurements, the B254 blade revealed the highest values. Although this blade had the longest crack with a crack length of 6.8 mm, the high nonlinearities can have various causes. Compared to the plate metal samples, the position of the cracks on the blades is variable. The crack of the blades B046 is only 5 mm behind the sensors, and the distance increases accordingly up to blade B286. In addition, real cracks behave differently, and many factors can lead to an increase of nonlinearities. The decisive factor is whether the crack is closed or open, and it is furthermore crucial how the asperities of the crack topography are in contact and how much of them are in interaction [39,40].

## 6. Conclusions

A comparison of various nonlinear ultrasonic wave modulation techniques was presented. The tests were conducted on metal plates, with artificially generated cracks, and on turbine blades with real cracks.

When using one excitation frequency, the results for both the plate samples and turbine blades were not convincing and showed that selecting the appropriate fundamental frequency is crucial to the success of the measurement.

The parallel excitation with two frequencies provides a total of 12 nonlinearity parameters to evaluate the components, and the new *N_index_* values were used as a comparison with the other excitation techniques. For the plate samples, some nonlinearity parameters revealed a clear increase depending on the crack size. With the two-frequency excitation with a linearly increasing signal, the informative value of the failures had not improved in comparison.

The four-frequency excitation was analytically solved using the extended one-dimensional wave equation. A total of 64 individual nonlinearity parameters were derived. Furthermore, the four fundamental frequencies were chosen so that as many different fundamental frequencies, harmonic frequencies, and sidebands as possible meet at one frequency. The concept of grouping nonlinear parameters simplifies the evaluation with a total of 30 nonlinearity parameters, which facilitates identifying a defect in the material. This behaviour and advantages were proven experimentally. The *N_index_* values of these measurements demonstrated behaviour as a function of the crack size. It was shown that, especially with the quasi-chaotic frequency evaluation, detecting the failure is possible without calculating dispersion curves.

The use of four fundamental frequencies offers clear advantages in detecting defects in materials by pairing with the frequency grouping method.

## Figures and Tables

**Table 1:** Nonlinearity parameter of quadruple-excitation.

Frequency	Nonlinearity Parameter
$f_{n}$ ^1^	$\gamma_{fn}\propto \frac{A_{f1+f2+f3+f4}}{A_{n}{}^{3}+A_{n}\left(\sum_{i=1}^{n-1}A_{i}{}^{2}+\sum_{i=n+1}^{4}A_{i}{}^{2}\right)}$
$2f_{n}$ ^1^	$\beta_{2fn}\propto \frac{A_{f1+f2+f3+f4}}{A_{n}{}^{2}}$
$3f_{n}$ ^1^	$\beta_{3fn}\propto \frac{A_{f1+f2+f3+f4}}{A_{n}{}^{3}}$
$f_{n}\pm f_{m}$ ^1,2^	$\beta_{fn\pm fm}\propto \frac{A_{f1+f2+f3+f4}}{A_{n}A_{m}}$
$2f_{n}\pm f_{m}$ ^1,3^	$\gamma_{2fn\pm fm}\propto \frac{A_{f1+f2+f3+f4}}{A_{n}{}^{2}A_{m}}$
$f_{n}+f_{m}\pm f_{p}$ ^1,3^	$\gamma_{f_{n}+f_{m}\pm f_{p}}\propto \frac{A_{f1+f2+f3+f4}}{A_{m}A_{n}A_{p}}$

^1^ n, m, p∈{1, 2, 3, 4}, ^2^ n>m, ^3^ n≠m≠p.

**Table 2:** Nonlinearity parameter group 1, *δ*_*G*1_.

Group	$A_{x}$	Frequency [MHz]	$\Gamma_{f/h/s_{x}}$	Nonlinearity Parameter Group $\delta_{G}$
1	1	2	2*f*_1_ − *f*_4_	$\delta_{G1}^{1}=\frac{A_{f1+f2+f3+f4}}{A_{1}{}^{2}A_{4}}$
	1	4	*f*_4_ − *f*_1_	$\delta_{G1}^{2}=\frac{A_{f1+f2+f3+f4}}{A_{4}A_{1}}$
	1	12	2*f*_1_	$\delta_{G1}^{3}=\frac{A_{f1+f2+f3+f4}}{A_{1}{}^{2}}$
	1	13.3	*f*_2_ + *f*_1_	$\delta_{G1}^{4}=\frac{A_{f1+f2+f3+f4}}{A_{2}A_{1}}$
	1	14	2*f*_4_ − *f*_1_	$\delta_{G1}^{5}=\frac{A_{f1+f2+f3+f4}}{A_{4}{}^{2}A_{1}}$
	1	18	3*f*_1_	$\delta_{G1}^{6}=\frac{A_{f1+f2+f3+f4}}{A_{1}{}^{3}}$
	1	18.7	*f*_4_ + *f*_3_	$\delta_{G1}^{7}=\frac{A_{f1+f2+f3+f4}}{A_{4}A_{3}}$
	1	19.3	2*f*_1_ + *f*_2_	$\delta_{G1}^{8}=\frac{A_{f1+f2+f3+f4}}{A_{1}{}^{2}A_{2}}$
	1	20	2*f*_4_	$\delta_{G1}^{9}=\frac{A_{f1+f2+f3+f4}}{A_{4}{}^{2}}$
	1	28.7	2*f*_4_ + *f*_3_	$\delta_{G1}^{10}=\frac{A_{f1+f2+f3+f4}}{A_{4}{}^{2}A_{3}}$
	1	30	3*f*_4_	$\delta_{G1}^{11}=\frac{A_{f1+f2+f3+f4}}{A_{4}{}^{3}}$

**Table 3:** Nonlinearity parameter group 2, *δ*_*G*2_.

Group	$A_{x}$	Frequency [MHz]	$\Gamma_{f/h/s_{x}}$	Nonlinearity Parameter Group $\delta_{G}$
2	1	2.6	*f*_3_ − *f*_1_	$\delta_{G2}^{1}=\frac{A_{f1+f2+f3+f4}}{A_{3}A_{1}+A_{4}A_{2}}$
	2		*f*_4_ − *f*_2_	
	1	3.3	*f*_1_ + *f*_2_ − *f*_4_	$\delta_{G2}^{2}=\frac{A_{f1+f2+f3+f4}}{A_{1}A_{2}A_{4}+A_{1}{}^{2}A_{3}}$
	2		2*f*_1_ − *f*_3_	
	1	12.6	*f*_3_ + *f*_4_ − *f*_1_	$\delta_{G2}^{3}=\frac{A_{f1+f2+f3+f4}}{A_{3}A_{4}A_{1}+A_{4}{}^{2}A_{2}}$
	2		2*f*_4_ − *f*_2_	
	1	14.7	2*f*_2_	$\delta_{G2}^{4}=\frac{A_{f1+f2+f3+f4}}{A_{2}{}^{2}+A_{3}A_{1}}$
	2		*f*_3_ + *f*_1_	
	1	16	*f*_4_ + *f*_1_	$\delta_{G2}^{5}=\frac{A_{f1+f2+f3+f4}}{A_{4}A_{1}+A_{3}A_{2}}$
	2		*f*_3_ + *f*_2_	
	1	17.3	2*f*_3_	$\delta_{G2}^{6}=\frac{A_{f1+f2+f3+f4}}{A_{3}{}^{2}+A_{4}A_{2}}$
	2		*f*_4_ + *f*_2_	
	1	20.7	2*f*_1_ + *f*_3_	$\delta_{G2}^{7}=\frac{A_{f1+f2+f3+f4}}{A_{1}{}^{2}A_{3}+A_{2}{}^{2}A_{1}}$
	2		2*f*_2_ + *f*_1_	
	1	27.3	2*f*_3_ + *f*_4_	$\delta_{G2}^{8}=\frac{A_{f1+f2+f3+f4}}{A_{3}{}^{2}A_{4}+A_{4}{}^{2}A_{2}}$
	2		2*f*_4_ + *f*_2_	

**Table 4:** Nonlinearity parameter group 3, *δ*_*G*3_.

Group	$A_{x}$	Frequency [MHz]	$\Gamma_{f/h/s_{x}}$	Nonlinearity Parameter Group $\delta_{G}$
3	1	1.3	*f*_2_ − *f*_1_	$\delta_{G3}^{1}=\frac{A_{f1+f2+f3+f4}}{A_{2}A_{1}+A_{3}A_{2}+A_{4}A_{3}}$
	2		*f*_3_ − *f*_2_	
	3		*f*_4_ − *f*_3_	
	1	6	*f*_1_	$\delta_{G3}^{2}=\frac{A_{f1+f2+f3+f4}}{A_{1}{}^{3}+A_{1}A_{2}{}^{2}+A_{1}A_{3}{}^{2}+A_{1}A_{4}{}^{2}+A_{2}{}^{2}A_{3}+A_{2}A_{3}A_{4}}$
	2		2*f*_2_ − *f*_3_	
	3		*f*_2_ + *f*_3_ − *f*_4_	
	1	10	*f*_4_	$\delta_{G3}^{3}=\frac{A_{f1+f2+f3+f4}}{A_{4}{}^{3}+A_{1}{}^{2}A_{4}+A_{2}{}^{2}A_{4}+A_{3}{}^{2}A_{4}+A_{3}{}^{2}A_{2}+A_{2}A_{3}A_{1}}$
	2		2*f*_3_ − *f*_2_	
	3		*f*_2_ + *f*_3_ − *f*_1_	
	1	22	3*f*_2_	$\delta_{G3}^{4}=\frac{A_{f1+f2+f3+f4}}{A_{2}{}^{3}+A_{1}{}^{2}A_{4}+A_{1}A_{2}A_{3}}$
	2		2*f*_1_ + *f*_4_	
	3		*f*_1_ + *f*_2_ + *f*_3_	
	1	23.3	2*f*_2_ + *f*_3_	$\delta_{G3}^{5}=\frac{A_{f1+f2+f3+f4}}{A_{2}{}^{2}A_{3}+A_{3}{}^{2}A_{1}+A_{1}A_{2}A_{4}}$
	2		2*f*_3_ + *f*_1_	
	3		*f*_1_ + *f*_2_ + *f*_4_	
	1	24.7	2*f*_2_ + *f*_4_	$\delta_{G3}^{6}=\frac{A_{f1+f2+f3+f4}}{A_{2}{}^{2}A_{4}+A_{3}{}^{2}A_{2}+A_{1}A_{3}A_{4}}$
	2		2*f*_3_ + *f*_2_	
	3		*f*_1_ + *f*_3_ + *f*_4_	
	1	26	3*f*_3_	$\delta_{G3}^{7}=\frac{A_{f1+f2+f3+f4}}{A_{3}{}^{3}+A_{4}{}^{2}A_{1}+A_{2}A_{3}A_{4}}$
	2		2*f*_4_ + *f*_1_	
	3		*f*_2_ + *f*_3_ + *f*_4_	

**Table 5:** Nonlinearity parameter group 4, *δ*_*G*4_.

Group	$A_{x}$	Frequency [MHz]	$\Gamma_{f/h/s_{x}}$	Nonlinearity Parameter Group $\delta_{G}$
4	1	4.6	2*f*_2_ − *f*_4_	$\delta_{G4}^{1}=\frac{A_{f1+f2+f3+f4}}{A_{2}{}^{2}A_{4}+A_{1}A_{2}A_{3}+A_{1}A_{3}A_{4}+A_{1}{}^{2}A_{2}}$
	2		*f*_1_ + *f*_2_ − *f*_3_	
	3		*f*_1_ + *f*_3_ − *f*_4_	
	4		2*f*_1_ − *f*_2_	
	1	7.3	2*f*_3_ − *f*_4_	$\delta_{G4}^{2}=\frac{A_{f1+f2+f3+f4}}{A_{3}{}^{2}A_{4}+A_{2}{}^{3}+A_{1}{}^{2}A_{2}+A_{2}A_{3}{}^{2}+A_{2}A_{4}{}^{2}+A_{1}A_{3}A_{2}+A_{1}A_{4}A_{3}}$
	2		*f*_2_	
	3		*f*_1_ + *f*_3_ − *f*_2_	
	4		*f*_1_ + *f*_4_ − *f*_3_	
	1	8.7	*f*_3_	$\delta_{G4}^{3}=\frac{A_{f1+f2+f3+f4}}{A_{3}{}^{3}+A_{1}{}^{2}A_{3}+A_{2}{}^{2}A_{3}+A_{3}A_{4}{}^{2}+A_{2}{}^{2}A_{1}+A_{2}A_{4}A_{3}+A_{1}A_{4}A_{2}}$
	2		2*f*_2_ − *f*_1_	
	3		*f*_2_ + *f*_4_ − *f*_3_	
	4		*f*_1_ + *f*_4_ − *f*_2_	
	1	11.3	2*f*_3_ − *f*_1_	$\delta_{G4}^{4}=\frac{A_{f1+f2+f3+f4}}{A_{3}{}^{2}A_{1}+A_{2}A_{4}A_{1}+A_{3}A_{4}A_{2}+A_{4}{}^{2}A_{3}}$
	2		*f*_2_ + *f*_4_ − *f*_1_	
	3		*f*_3_ + *f*_4_ − *f*_2_	
	4		2*f*_4_ − *f*_3_	

**Table 6:** Signal grouping.

Group	*Γ*_*f*/*h*/*s*_	Frequency [MHz]
-	2*f*_1_ − *f*_2_	0
1	*f*_1_	5
	*f*_2_ − *f*_1_	5
2	*f*_2_	10
	2*f*_1_	10
3	3*f*_1_	15
	*f*_2_ + *f*_1_	15
	2*f*_2_ − *f*_1_	15
4	2*f*_2_	20
	2*f*_1_ + *f*_2_	20
-	2*f*_2_ + *f*_1_	25
-	3*f*_2_	30

**Table 7:** Overview—turbine blades.

Blade	Crack Position [Cooling Air Outlet Chamber (Figure 26)]	Crack Length [mm]
B292	-	-
B046	2	6.0
B254	3	6.8
B286	6	6.1

## Appendix A

This section includes the further derivation for the solution of the one-dimensional wave equation with four excitation frequencies up to the third-order of nonlinearity (Section 2.1).

Substituting Equation (7) in the right side of Equation (5) with the second-order nonlinearity parameter *β* and making further transformations with trigonometric formulas lead to:

(A1) $$\begin{array}{c} \frac{\partial^{2}u}{\partial t^{2}}-c^{2}\frac{\partial^{2}u}{\partial x^{2}}=\\ c^{2}\beta (\begin{array}{c} -\frac{1}{2}A_{1}^{2}k_{f1}^{3}sin\left[k_{f1}\left(x-ct\right)\right]\\ +\frac{1}{2}A_{2}^{2}k_{f2}^{3}sin\left[k_{f2}\left(x-ct\right)\right]\\ -\frac{1}{2}A_{3}^{2}k_{f3}^{3}sin\left[k_{f3}\left(x-ct\right)\right]\\ +\frac{1}{2}A_{4}^{2}k_{f4}^{3}sin\left[k_{f4}\left(x-ct\right)\right]\\ -\frac{1}{2}A_{1}A_{2}k_{f1}k_{f2}(k_{f2}-k_{f1})cos\left[(k_{f2}-k_{f1})\left(x-ct\right)\right]\\ -\frac{1}{2}A_{1}A_{2}k_{f1}k_{f2}(k_{f2}+k_{f1})cos\left[(k_{f2}+k_{f1})\left(x-ct\right)\right]\\ -\frac{1}{2}A_{1}A_{3}k_{f1}k_{f3}(k_{f3}-k_{f1})sin\left[(k_{f3}-k_{f1})\left(x-ct\right)\right]\\ -\frac{1}{2}A_{1}A_{3}k_{f1}k_{f3}(k_{f3}+k_{f1})sin\left[(k_{f3}+k_{f1})\left(x-ct\right)\right]\\ -\frac{1}{2}A_{1}A_{4}k_{f1}k_{f4}(k_{f4}-k_{f1})cos\left[(k_{f4}-k_{f1})\left(x-ct\right)\right]\\ -\frac{1}{2}A_{1}A_{4}k_{f1}k_{f4}(k_{f4}+k_{f1})cos\left[(k_{f4}-k_{f1})\left(x+ct\right)\right]\\ +\frac{1}{2}A_{2}A_{3}k_{f2}k_{f3}(k_{f3}-k_{f2})cos\left[(k_{f3}-k_{f2})\left(x-ct\right)\right]\\ -\frac{1}{2}A_{2}A_{3}k_{f2}k_{f3}(k_{f3}+k_{f2})cos\left[(k_{f3}+k_{f2})\left(x-ct\right)\right]\\ +\frac{1}{2}A_{2}A_{4}k_{f2}k_{f4}(k_{f4}-k_{f2})sin\left[(k_{f4}-k_{f2})\left(x-ct\right)\right]\\ +\frac{1}{2}A_{2}A_{4}k_{f2}k_{f4}(k_{f4}+k_{f2})sin\left[(k_{f4}+k_{f2})\left(x-ct\right)\right]\\ -\frac{1}{2}A_{3}A_{4}k_{f3}k_{f4}(k_{f4}-k_{f3})cos\left[(k_{f4}-k_{f3})\left(x-ct\right)\right]\\ -\frac{1}{2}A_{3}A_{4}k_{f3}k_{f4}(k_{f4}+k_{f3})cos\left[(k_{f4}+k_{f3})\left(x-ct\right)\right] \end{array}). \end{array}$$

Based on this expression and according to [17], the following solution results for $u^{\left(2\right)}$:

(A2) $$\begin{array}{c} u^{\left(2\right)}=-\frac{\beta A_{1}^{2}k_{f1}^{2}}{8}\cos\left[2k_{f1}\left(x-ct\right)\right]x+\frac{\beta A_{2}^{2}k_{f2}^{2}}{8}\cos\left[2k_{f2}\left(x-ct\right)\right]x\\ -\frac{\beta A_{3}^{2}k_{f3}^{2}}{8}\cos\left[2k_{f3}\left(x-ct\right)\right]x+\frac{\beta A_{4}^{2}k_{f4}^{2}}{8}\cos\left[2k_{f4}\left(x-ct\right)\right]x\\ +\frac{\beta A_{1}A_{2}k_{f1}k_{f2}}{4}\sin\left[\left(k_{f2}-k_{f1}\right)\left(x-ct\right)\right]x+\frac{\beta A_{1}A_{2}k_{f1}k_{f2}}{4}\sin\left[\left(k_{f2}+k_{f1}\right)\left(x-ct\right)\right]x\\ +\frac{\beta A_{1}A_{3}k_{f1}k_{f3}}{4}\cos\left[\left(k_{f3}-k_{f1}\right)\left(x-ct\right)\right]x-\frac{\beta A_{1}A_{3}k_{f1}k_{f3}}{4}\cos\left[\left(k_{f3}+k_{f1}\right)\left(x-ct\right)\right]x\\ -\frac{\beta A_{1}A_{4}k_{f1}k_{f4}}{4}\sin\left[\left(k_{f4}-k_{f1}\right)\left(x-ct\right)\right]x-\frac{\beta A_{1}A_{4}k_{f1}k_{f4}}{4}\sin\left[\left(k_{f4}+k_{f1}\right)\left(x-ct\right)\right]x\\ -\frac{\beta A_{2}A_{3}k_{f2}k_{f3}}{4}\sin\left[\left(k_{f3}-k_{f2}\right)\left(x-ct\right)\right]x+\frac{\beta A_{2}A_{3}k_{f2}k_{f3}}{4}\sin\left[\left(k_{f3}+k_{f2}\right)\left(x-ct\right)\right]x\\ -\frac{\beta A_{2}A_{4}k_{f2}k_{f4}}{4}\cos\left[\left(k_{f4}-k_{f2}\right)\left(x-ct\right)\right]x+\frac{\beta A_{2}A_{4}k_{f2}k_{f4}}{4}\cos\left[\left(k_{f4}+k_{f2}\right)\left(x-ct\right)\right]x\\ +\frac{\beta A_{3}A_{4}k_{f3}k_{f4}}{4}\sin\left[\left(k_{f4}-k_{f3}\right)\left(x-ct\right)\right]x+\frac{\beta A_{3}A_{4}k_{f3}k_{f4}}{4}\sin\left[\left(k_{f4}+k_{f3}\right)\left(x-ct\right)\right]x. \end{array}$$

The solution of Equation (5) should now be extended by the cubic nonlinearity parameter *γ* to obtain an encompassing description of the nonlinearity parameters. Substituting Equation (7) into the right side of Equation (5) in the third-order nonlinearity parameter *γ* gives:

(A3) $$\begin{array}{c} \frac{\partial^{2}u}{\partial t^{2}}-c^{2}\frac{\partial^{2}u}{\partial x^{2}}=\\ \frac{c^{2}\gamma }{2}[{\left(\begin{array}{c} A_{1}sin\left[k_{f1}\left(x-ct\right)\right]+A_{2}cos\left[k_{f2}\left(x-ct\right)\right]+\\ A_{3}sin\left[k_{f3}\left(x-ct\right)\right]+A_{4}cos\left[k_{f4}\left(x-ct\right)\right] \end{array}\right)}^{2}\\ \begin{array}{c} \left(\begin{array}{c} -A_{1}k_{f1}{}^{2}sin\left[k_{f1}\left(x-ct\right)\right]-A_{2}k_{f2}{}^{2}cos\left[k_{2}\left(x-ct\right)\right]\\ -A_{3}k_{f3}{}^{2}sin\left[k_{f3}\left(x-ct\right)\right]-A_{4}k_{f4}{}^{2}cos\left[k_{4}\left(x-ct\right)\right] \end{array}\right) \end{array}]. \end{array}$$

Transformation with binominal and trigonometric formulas leads to:

(A4) $$\frac{\partial^{2}u}{\partial t^{2}}-c^{2}\frac{\partial^{2}u}{\partial x^{2}}=\frac{c^{2}\gamma }{2}(\begin{array}{c} -\frac{1}{2}\left(A_{1}A_{2}{}^{2}k_{f1}{}^{2}k_{f2}{}^{2}+A_{1}A_{4}{}^{2}k_{f1}{}^{2}k_{f4}{}^{2}+A_{1}A_{3}{}^{2}k_{f1}{}^{2}k_{f3}{}^{2}+\frac{1}{2}A_{1}{}^{3}k_{f1}{}^{4}\right)sin\left[k_{f1}\left(x-ct\right)\right]\\ -\frac{1}{2}\left(A_{1}{}^{2}A_{2}k_{f1}{}^{2}k_{f2}{}^{2}+A_{2}A_{4}{}^{2}k_{f2}{}^{2}k_{f4}{}^{2}+A_{2}A_{3}{}^{2}k_{f2}{}^{2}k_{f3}{}^{2}+\frac{1}{2}A_{2}{}^{3}k_{f2}{}^{4}\right)sin\left[k_{f2}\left(x-ct\right)\right]\\ -\frac{1}{2}\left(A_{1}{}^{2}A_{3}k_{f1}{}^{2}k_{f3}{}^{2}+A_{2}{}^{2}A_{3}k_{f2}{}^{2}k_{f3}{}^{2}+A_{3}A_{4}{}^{2}k_{f3}{}^{2}k_{f4}{}^{2}+\frac{1}{2}A_{3}{}^{3}k_{f3}{}^{4}\right)sin\left[k_{f3}\left(x-ct\right)\right]\\ -\frac{1}{2}\left(A_{1}{}^{2}A_{4}k_{f1}{}^{2}k_{f4}{}^{2}+A_{2}{}^{2}A_{4}k_{f2}{}^{2}k_{f4}{}^{2}+A_{3}{}^{2}A_{4}k_{f3}{}^{2}k_{f4}{}^{2}+\frac{1}{2}A_{4}{}^{3}k_{f4}{}^{4}\right)sin\left[k_{f4}\left(x-ct\right)\right]\\ -\frac{1}{4}A_{1}{}^{3}k_{f1}{}^{4}sin\left[3k_{f1}\left(x-ct\right)\right]+\frac{1}{4}A_{2}{}^{3}k_{f2}{}^{4}cos\left[3k_{f2}\left(x-ct\right)\right]\\ -\frac{1}{4}A_{3}{}^{3}k_{f3}{}^{4}sin\left[3k_{f3}\left(x-ct\right)\right]+\frac{1}{4}A_{4}{}^{3}k_{f4}{}^{4}cos\left[3k_{f4}\left(x-ct\right)\right]\\ -\frac{1}{2}A_{1}{}^{2}A_{2}\left(k_{f1}{}^{3}k_{f2}+\frac{1}{2}k_{f1}{}^{2}k_{f2}{}^{2}\right)cos\left[(2k_{f1}+k_{f2})\left(x-ct\right)\right]\\ +\frac{1}{2}A_{1}{}^{2}A_{2}\left(k_{f1}{}^{3}k_{f2}-\frac{1}{2}k_{f1}{}^{2}k_{f2}{}^{2}\right)cos\left[(2k_{f1}-k_{f2})\left(x-ct\right)\right]\\ -\frac{1}{2}A_{1}{}^{2}A_{3}\left(\frac{1}{2}k_{f1}{}^{2}k_{f3}{}^{2}+k_{f1}{}^{3}k_{f3}\right)sin\left[(2k_{f1}+k_{f3})\left(x-ct\right)\right]\\ +\frac{1}{2}A_{1}{}^{2}A_{3}\left(\frac{1}{2}k_{f1}{}^{2}k_{f3}{}^{2}-k_{f1}{}^{3}k_{f3}\right)sin\left[(2k_{f1}-k_{f3})\left(x-ct\right)\right]\\ -\frac{1}{2}A_{1}{}^{2}A_{4}\left(k_{f1}{}^{3}k_{f4}+\frac{1}{2}k_{f1}{}^{2}k_{f4}{}^{2}\right)cos\left[(2k_{f1}+k_{f4})\left(x-ct\right)\right]\\ +\frac{1}{2}A_{1}{}^{2}A_{4}\left(k_{f1}{}^{3}k_{f4}-\frac{1}{2}k_{f1}{}^{2}k_{f4}{}^{2}\right)cos\left[(2k_{f1}-k_{f4})\left(x-ct\right)\right]\\ +\frac{1}{2}A_{1}A_{2}{}^{2}\left(\frac{1}{2}k_{f1}{}^{2}k_{f2}{}^{2}+k_{f1}k_{f2}{}^{3}\right)sin\left[(2k_{f2}+k_{f1})\left(x-ct\right)\right]\\ +\frac{1}{2}A_{1}A_{2}{}^{2}\left(k_{f1}k_{f2}{}^{3}-\frac{1}{2}k_{f1}{}^{2}k_{f2}{}^{2}\right)sin\left[(2k_{f2}-k_{f1})\left(x-ct\right)\right]\\ +\frac{1}{2}A_{2}{}^{2}A_{3}\left(k_{f2}{}^{3}k_{f3}+\frac{1}{2}k_{f2}{}^{2}k_{f3}{}^{2}\right)sin\left[(2k_{f2}+k_{f3})\left(x-ct\right)\right]\\ +\frac{1}{2}A_{2}{}^{2}A_{3}\left(k_{f2}{}^{3}k_{f3}-\frac{1}{2}k_{f2}{}^{2}k_{f3}{}^{2}\right)sin\left[(2k_{f2}-k_{f3})\left(x-ct\right)\right]\\ +\frac{1}{4}A_{2}{}^{2}A_{4}k_{f2}{}^{2}k_{f4}{}^{2}cos\left[(2k_{f2}+k_{f4})\left(x-ct\right)\right]\\ +\frac{1}{4}A_{2}{}^{2}A_{4}k_{f2}{}^{2}k_{f4}{}^{2}cos\left[(2k_{f2}-k_{f4})\left(x-ct\right)\right]\\ -\frac{1}{2}A_{1}A_{3}{}^{2}\left(\frac{1}{2}k_{f1}{}^{2}k_{f3}{}^{2}+k_{f1}k_{f3}{}^{3}\right)sin\left[(2k_{f3}+k_{f1})\left(x-ct\right)\right]\\ +\frac{1}{2}A_{1}A_{3}{}^{2}\left(\frac{1}{2}k_{f1}{}^{2}k_{f3}{}^{2}-k_{f1}k_{f3}{}^{3}\right)sin\left[(2k_{f3}-k_{f1})\left(x-ct\right)\right]\\ -\frac{1}{2}A_{1}A_{3}{}^{2}\left(\frac{1}{2}k_{f2}{}^{2}k_{f3}{}^{2}+k_{f2}k_{f3}{}^{3}\right)cos\left[(2k_{f3}+k_{f2})\left(x-ct\right)\right]\\ +\frac{1}{2}A_{2}A_{3}{}^{2}\left(k_{f2}k_{f3}{}^{3}-\frac{1}{2}k_{f2}{}^{2}k_{f3}{}^{2}\right)cos\left[(2k_{f3}-k_{f2})\left(x-ct\right)\right]\\ -\frac{1}{2}A_{3}{}^{2}A_{4}\left(k_{f3}{}^{3}k_{f4}+\frac{1}{2}k_{f3}{}^{2}k_{f4}{}^{2}\right)cos\left[(2k_{f3}+k_{f4})\left(x-ct\right)\right]\\ +\frac{1}{2}A_{3}{}^{2}A_{4}\left(k_{f3}{}^{3}k_{f4}-\frac{1}{2}k_{f3}{}^{2}k_{f4}{}^{2}\right)cos\left[(2k_{f3}-k_{f4})\left(x-ct\right)\right]\\ +\frac{1}{2}A_{1}A_{4}{}^{2}\left(\frac{1}{2}k_{f1}{}^{2}k_{f4}{}^{2}+k_{f1}k_{f4}{}^{3}\right)sin\left[(2k_{f4}+k_{f1})\left(x-ct\right)\right]\\ +\frac{1}{2}A_{1}A_{4}{}^{2}\left(k_{f1}k_{f4}{}^{3}-\frac{1}{2}k_{f1}{}^{2}k_{f4}{}^{2}\right)sin\left[(2k_{f4}-k_{f1})\left(x-ct\right)\right]\\ +\frac{1}{4}A_{2}A_{4}{}^{2}k_{f2}{}^{2}k_{f4}{}^{2}cos\left[(2k_{f4}+k_{f2})\left(x-ct\right)\right]\\ +\frac{1}{4}A_{2}A_{4}{}^{2}k_{f2}{}^{2}k_{f4}{}^{2}cos\left[(2k_{f4}-k_{f2})\left(x-ct\right)\right]\\ +\frac{1}{2}A_{3}A_{4}{}^{2}\left(\frac{1}{2}k_{f3}{}^{2}k_{f4}{}^{2}+k_{f3}k_{f4}{}^{3}\right)sin\left[(2k_{f4}+k_{f3})\left(x-ct\right)\right]\\ +\frac{1}{2}A_{3}A_{4}{}^{2}\left(k_{f3}k_{f4}{}^{3}-\frac{1}{2}k_{f3}{}^{2}k_{f4}{}^{2}\right)sin\left[(2k_{f4}-k_{f3})\left(x-ct\right)\right]\\ -\frac{1}{2}A_{1}A_{2}A_{3}\left(k_{f1}{}^{2}k_{f2}k_{f3}+k_{f1}k_{f2}{}^{2}k_{f3}+k_{f1}k_{f2}k_{f3}{}^{2}\right)cos\left[(k_{f1}+k_{f2}+k_{f3})\left(x-ct\right)\right]\\ -\frac{1}{2}A_{1}A_{2}A_{3}\left(k_{f1}{}^{2}k_{f2}k_{f3}+k_{f1}k_{f2}{}^{2}k_{f3}-k_{f1}k_{f2}k_{f3}{}^{2}\right)cos\left[(k_{f1}+k_{f2}-k_{f3})\left(x-ct\right)\right]\\ -\frac{1}{2}A_{1}A_{3}A_{4}\left(k_{f1}{}^{2}k_{f3}k_{f4}+k_{f1}k_{f3}{}^{2}k_{f4}+k_{f1}k_{f3}k_{f4}{}^{2}\right)cos\left[(k_{f1}+k_{f3}+k_{f4})\left(x-ct\right)\right]\\ +\frac{1}{2}A_{1}A_{3}A_{4}\left(k_{f1}{}^{2}k_{f3}k_{f4}+k_{f1}k_{f3}{}^{2}k_{f4}-k_{f1}k_{f3}k_{f4}{}^{2}\right)cos\left[(k_{f1}+k_{f3}-k_{f4})\left(x-ct\right)\right]\\ +\frac{1}{2}A_{1}A_{2}A_{4}\left(k_{f1}{}^{2}k_{f2}k_{f4}+k_{f1}k_{f2}k_{f4}{}^{2}\right)sin\left[(k_{f1}+k_{f2}+k_{f4})\left(x-ct\right)\right]\\ +\frac{1}{2}A_{1}A_{2}A_{4}\left(k_{f1}{}^{2}k_{f2}k_{f4}-k_{f1}k_{f2}k_{f4}{}^{2}\right)sin\left[(k_{f1}+k_{f2}-k_{f4})\left(x-ct\right)\right]\\ +\frac{1}{2}A_{1}A_{2}A_{3}\left(k_{f1}{}^{2}k_{f2}k_{f3}-k_{f1}k_{f2}{}^{2}k_{f3}-k_{f1}k_{f2}k_{f3}{}^{2}\right)cos\left[(k_{f2}+k_{f3}-k_{f1})\left(x-ct\right)\right]\\ +\frac{1}{2}A_{1}A_{3}A_{4}\left(k_{f1}k_{f3}{}^{2}k_{f4}-k_{f1}{}^{2}k_{f3}k_{f4}-k_{f1}k_{f2}k_{f4}{}^{2}\right)cos\left[(k_{f1}+k_{f4}-k_{f3})\left(x-ct\right)\right]\\ +\frac{1}{2}A_{1}A_{2}A_{4}\left(k_{f1}{}^{2}k_{f3}k_{f4}-k_{f1}k_{f3}{}^{2}k_{f4}-k_{f1}k_{f3}k_{f4}{}^{2}\right)cos\left[(k_{f3}+k_{f4}-k_{f1})\left(x-ct\right)\right]\\ +\frac{1}{2}A_{1}A_{2}A_{4}\left(k_{f1}k_{f2}{}^{2}k_{f4}+k_{f1}k_{f2}k_{f4}{}^{2}\right)sin\left[(k_{f2}+k_{f4}-k_{f1})\left(x-ct\right)\right]\\ +\frac{1}{2}A_{1}A_{2}A_{3}\left(k_{f1}k_{f2}k_{f3}{}^{2}-k_{f1}k_{f2}{}^{2}k_{f3}+k_{f1}{}^{2}k_{f2}k_{f3}\right)cos\left[(k_{f1}+k_{f3}-k_{f2})\left(x-ct\right)\right]\\ +\frac{1}{2}A_{2}A_{3}A_{4}\left(k_{f2}{}^{2}k_{f3}k_{f4}-k_{f2}k_{f3}k_{f4}{}^{2}\right)sin\left[(k_{f2}+k_{f3}+k_{f4})\left(x-ct\right)\right]\\ +\frac{1}{2}A_{2}A_{3}A_{4}\left(k_{f2}{}^{2}k_{f3}k_{f4}-k_{f2}k_{f3}k_{f4}{}^{2}\right)sin\left[(k_{f2}+k_{f4}-k_{f3})\left(x-ct\right)\right]\\ -\frac{1}{2}A_{2}A_{3}A_{4}\left(k_{f2}{}^{2}k_{f3}k_{f4}+k_{f2}k_{f3}k_{f4}{}^{2}\right)sin\left[(k_{f2}+k_{f3}-k_{f4})\left(x-ct\right)\right]\\ +\frac{1}{2}A_{2}A_{3}A_{4}\left(k_{f2}{}^{2}k_{f3}k_{f4}+k_{f2}k_{f3}k_{f4}{}^{2}\right)sin\left[(k_{f3}+k_{f4}-k_{f2})\left(x-ct\right)\right]\\ -\frac{1}{2}A_{1}A_{2}A_{4}\left(k_{f1}k_{f2}k_{f4}{}^{2}\right)sin\left[(k_{f1}+k_{f4}-k_{f2})\left(x-ct\right)\right] \end{array}).$$

Based on [17], this results in the following solution:

(A5) $$\begin{array}{c} u^{\left(3\right)}=+\left(\frac{\gamma (A_{1}A_{2}{}^{2}k_{f1}k_{f2}{}^{2}+A_{1}A_{4}{}^{2}k_{f1}k_{f4}{}^{2}+A_{1}A_{3}{}^{2}k_{f1}k_{f3}{}^{2}+\frac{1}{2}A_{1}{}^{3}k_{f1}{}^{4})}{8}\right)cos\left[k_{f1}\left(x-ct\right)\right]x\\ -\left(\frac{\gamma (A_{1}{}^{2}A_{2}k_{f1}{}^{2}k_{f2}+A_{2}A_{4}{}^{2}k_{f1}k_{f4}{}^{2}+A_{2}A_{3}{}^{2}k_{f2}k_{f3}{}^{2}+\frac{1}{2}A_{2}{}^{3}k_{f2}{}^{4})}{8}\right)sin\left[k_{f2}\left(x-ct\right)\right]x\\ +\left(\frac{\gamma (A_{1}{}^{2}A_{3}k_{f1}{}^{2}k_{f3}+A_{2}{}^{2}A_{3}k_{f2}{}^{2}k_{f3}+A_{3}A_{4}{}^{2}k_{f3}k_{f4}{}^{2}+\frac{1}{2}A_{3}{}^{3}k_{f3}{}^{4})}{8}\right)cos\left[k_{f3}\left(x-ct\right)\right]x\\ -\left(\frac{\gamma (A_{1}{}^{2}A_{4}k_{f1}{}^{2}k_{f4}+A_{2}{}^{2}A_{4}k_{f2}{}^{2}k_{f4}+A_{3}{}^{2}A_{4}k_{f3}{}^{2}k_{f4}+\frac{1}{2}A_{4}{}^{3}k_{f4}{}^{4})}{8}\right)sin\left[k_{f4}\left(x-ct\right)\right]x\\ +\frac{\gamma A_{1}{}^{3}k_{f1}{}^{3}}{48}cos\left[3k_{f1}\left(x-ct\right)\right]x+\frac{\gamma A_{2}{}^{3}k_{f2}{}^{3}}{48}sin\left[3k_{f2}\left(x-ct\right)\right]x\\ +\frac{\gamma A_{3}{}^{3}k_{f3}{}^{3}}{48}cos\left[3k_{f3}\left(x-ct\right)\right]x+\frac{\gamma A_{4}{}^{3}k_{f4}{}^{3}}{48}sin\left[3k_{f4}\left(x-ct\right)\right]x\\ -\left(\frac{\gamma A_{1}{}^{2}A_{2}\left(k_{f1}{}^{3}k_{f2}+\frac{1}{2}k_{f1}{}^{2}k_{f2}{}^{2}\right)}{8\left(2k_{f1}+k_{f2}\right)}\right)sin\left[\left(2k_{f1}+k_{f2}\right)\left(x-ct\right)\right]x+\left(\frac{\gamma A_{1}{}^{2}A_{2}\left(k_{f1}{}^{3}k_{f2}-\frac{1}{2}k_{f1}{}^{2}k_{f2}{}^{2}\right)}{8\left(2k_{f1}-k_{f2}\right)}\right)sin\left[\left(2k_{f1}-k_{f2}\right)\left(x-ct\right)\right]x\\ +\left(\frac{\gamma A_{1}{}^{2}A_{3}\left(\frac{1}{2}k_{f1}{}^{2}k_{f3}{}^{2}+k_{f1}{}^{3}k_{f3}\right)}{8\left(2k_{f1}+k_{f3}\right)}\right)cos\left[\left(2k_{f1}+k_{f3}\right)\left(x-ct\right)\right]x+\left(\frac{\gamma A_{1}{}^{2}A_{3}\left(\frac{1}{2}k_{f1}{}^{2}k_{f3}{}^{2}-k_{f1}{}^{3}k_{f3}\right)}{8\left(2k_{f1}-k_{f3}\right)}\right)cos\left[\left(2k_{f1}-k_{f3}\right)\left(x-ct\right)\right]x\\ -\left(\frac{\gamma A_{1}{}^{2}A_{4}\left(k_{f1}{}^{3}k_{f4}+\frac{1}{2}k_{f1}{}^{2}k_{f4}{}^{2}\right)}{8\left(2k_{f1}+k_{f4}\right)}\right)sin\left[\left(2k_{f1}+k_{f4}\right)\left(x-ct\right)\right]x+\left(\frac{\gamma A_{1}{}^{2}A_{4}\left(k_{f1}{}^{3}k_{f4}-\frac{1}{2}k_{f1}{}^{2}k_{f4}{}^{2}\right)}{8\left(2k_{f1}-k_{f4}\right)}\right)sin\left[\left(2k_{f1}-k_{f4}\right)\left(x-ct\right)\right]x\\ -\left(\frac{\gamma A_{1}A_{2}{}^{2}\left(\frac{1}{2}k_{f1}{}^{2}k_{f2}{}^{2}+k_{f1}k_{f2}{}^{3}\right)}{8\left(2k_{f2}+k_{f1}\right)}\right)cos\left[\left(2k_{f2}+k_{f1}\right)\left(x-ct\right)\right]x-\left(\frac{\gamma A_{1}A_{2}{}^{2}\left(\frac{1}{2}k_{f1}{}^{2}k_{f2}{}^{2}-k_{f1}k_{f2}{}^{3}\right)}{8\left(2k_{f2}-k_{f1}\right)}\right)cos\left[\left(2k_{f2}-k_{f1}\right)\left(x-ct\right)\right]x\\ -\left(\frac{\gamma A_{2}{}^{2}A_{3}\left(k_{f2}{}^{3}k_{f3}+\frac{1}{2}k_{f2}{}^{2}k_{f3}{}^{2}\right)}{8\left(2k_{f2}+k_{f3}\right)}\right)cos\left[\left(2k_{f2}+k_{f3}\right)\left(x-ct\right)\right]x-\left(\frac{\gamma A_{2}{}^{2}A_{3}\left(k_{f2}{}^{3}k_{f3}-\frac{1}{2}k_{f2}{}^{2}k_{f3}{}^{2}\right)}{8\left(2k_{f2}-k_{f3}\right)}\right)cos\left[\left(2k_{f2}-k_{f3}\right)\left(x-ct\right)\right]x\\ +\frac{\gamma A_{2}{}^{2}A_{4}k_{f2}{}^{2}k_{f4}{}^{2}}{16\left(2k_{f2}+k_{f4}\right)}sin\left[\left(2k_{f2}+k_{f4}\right)\left(x-ct\right)\right]x+\frac{\gamma A_{2}{}^{2}A_{4}k_{f2}{}^{2}k_{f4}{}^{2}}{16\left(2k_{f2}-k_{f4}\right)}sin\left[\left(2k_{f2}-k_{f4}\right)\left(x-ct\right)\right]x\\ +\left(\frac{\gamma A_{1}A_{3}{}^{2}\left(\frac{1}{2}k_{f1}{}^{2}k_{f3}{}^{2}+k_{f1}k_{f3}{}^{3}\right)}{8\left(2k_{f3}+k_{f1}\right)}\right)cos\left[\left(2k_{f3}+k_{f1}\right)\left(x-ct\right)\right]x+\left(\frac{\gamma A_{1}A_{3}{}^{2}\left(\frac{1}{2}k_{f1}{}^{2}k_{f3}{}^{2}-k_{f1}k_{f3}{}^{3}\right)}{8\left(2k_{f3}-k_{f1}\right)}\right)cos\left[\left(2k_{f3}-k_{f1}\right)\left(x-ct\right)\right]x\\ -\left(\frac{\gamma A_{2}A_{3}{}^{2}\left(\frac{1}{2}k_{f2}{}^{2}k_{f3}{}^{2}+k_{f2}k_{f3}{}^{3}\right)}{8\left(2k_{f3}+k_{f2}\right)}\right)sin\left[\left(2k_{f3}+k_{f2}\right)\left(x-ct\right)\right]x+\left(\frac{\gamma A_{2}A_{3}{}^{2}\left(\frac{1}{2}k_{f2}{}^{2}k_{f3}{}^{2}-k_{f2}k_{f3}{}^{3}\right)}{8\left(2k_{f3}-k_{f2}\right)}\right)sin\left[\left(2k_{f3}-k_{f2}\right)\left(x-ct\right)\right]x\\ -\left(\frac{\gamma A_{3}{}^{2}A_{4}\left(k_{f3}{}^{3}k_{f4}+\frac{1}{2}k_{f3}{}^{2}k_{f4}{}^{2}\right)}{8\left(2k_{f3}+k_{f4}\right)}\right)sin\left[\left(2k_{f3}+k_{f4}\right)\left(x-ct\right)\right]x+\left(\frac{\gamma A_{3}{}^{2}A_{4}\left(k_{f3}{}^{3}k_{f4}-\frac{1}{2}k_{f3}{}^{2}k_{f4}{}^{2}\right)}{8\left(2k_{f3}-k_{f4}\right)}\right)sin\left[\left(2k_{f3}-k_{f4}\right)\left(x-ct\right)\right]x\\ -\left(\frac{\gamma A_{1}A_{4}{}^{2}\left(\frac{1}{2}k_{f1}{}^{2}k_{f4}{}^{2}+k_{f1}k_{f4}{}^{3}\right)}{8\left(2k_{f4}+k_{f1}\right)}\right)cos\left[\left(2k_{f4}+k_{f1}\right)\left(x-ct\right)\right]x-\left(\frac{\gamma A_{1}A_{4}{}^{2}\left(\frac{1}{2}k_{f1}{}^{2}k_{f4}{}^{2}-k_{f1}k_{f4}{}^{3}\right)}{8\left(2k_{f4}-k_{f1}\right)}\right)cos\left[\left(2k_{f4}-k_{f1}\right)\left(x-ct\right)\right]x\\ +\frac{\gamma A_{2}A_{4}{}^{2}k_{f2}{}^{2}k_{f4}{}^{2}}{16\left(2k_{f4}+k_{f2}\right)}sin\left[\left(2k_{f4}+k_{f2}\right)\left(x-ct\right)\right]x+\frac{\gamma A_{2}A_{4}{}^{2}k_{f2}{}^{2}k_{f4}{}^{2}}{16\left(2k_{f4}-k_{f2}\right)}sin\left[\left(2k_{f4}-k_{f2}\right)\left(x-ct\right)\right]x\\ -\left(\frac{\gamma A_{3}A_{4}{}^{2}\left(\frac{1}{2}k_{f3}{}^{2}k_{f4}{}^{2}+k_{f3}k_{f4}{}^{3}\right)}{8\left(2k_{f4}+k_{f3}\right)}\right)cos\left[\left(2k_{f4}+k_{f3}\right)\left(x-ct\right)\right]x-\left(\frac{\gamma A_{3}A_{4}{}^{2}\left(k_{f3}k_{f4}{}^{3}-\frac{1}{2}k_{f3}{}^{2}k_{f4}{}^{2}\right)}{8\left(2k_{f4}-k_{f3}\right)}\right)cos\left[\left(2k_{f4}-k_{f3}\right)\left(x-ct\right)\right]x\\ -\left(\frac{\gamma A_{1}A_{2}A_{3}\left(k_{f1}{}^{2}k_{f2}k_{f3}+k_{f1}k_{f2}{}^{2}k_{f3}+k_{f1}k_{f2}k_{f3}{}^{2}\right)}{8\left(k_{f1}+k_{f2}+k_{f3}\right)}\right)sin\left[\left(k_{f1}+k_{f2}+k_{f3}\right)\left(x-ct\right)\right]x\\ -\left(\frac{\gamma A_{1}A_{2}A_{3}\left(k_{f1}{}^{2}k_{f2}k_{f3}+k_{f1}k_{f2}{}^{2}k_{f3}-k_{f1}k_{f2}k_{f3}{}^{2}\right)}{8\left(k_{f1}+k_{f2}-k_{f3}\right)}\right)sin\left[\left(k_{f1}+k_{f2}-k_{f3}\right)\left(x-ct\right)\right]x\\ -\left(\frac{\gamma A_{1}A_{3}A_{4}\left(k_{f1}{}^{2}k_{f3}k_{f4}+k_{f1}k_{f3}{}^{2}k_{f4}+k_{f1}k_{f3}k_{f4}{}^{2}\right)}{8\left(k_{f1}+k_{f3}+k_{f4}\right)}\right)sin\left[\left(k_{f1}+k_{f3}+k_{f4}\right)\left(x-ct\right)\right]x\\ +\left(\frac{\gamma A_{1}A_{2}4\left(k_{f1}{}^{2}k_{f3}k_{f4}+k_{f1}k_{f3}{}^{2}k_{f4}-k_{f1}k_{f3}k_{f4}{}^{2}\right)}{8\left(k_{f1}+k_{f3}-k_{f4}\right)}\right)sin\left[\left(k_{f1}+k_{f3}-k_{f4}\right)\left(x-ct\right)\right]x\\ -\left(\frac{\gamma A_{1}A_{2}A_{4}\left(k_{f1}k_{f2}{}^{2}k_{f4}+k_{f1}k_{f2}k_{f4}{}^{2}\right)}{8\left(k_{f1}+k_{f2}+k_{f4}\right)}\right)cos\left[\left(k_{f1}+k_{f2}+k_{f4}\right)\left(x-ct\right)\right]x\\ -\left(\frac{\gamma A_{1}A_{2}A_{4}\left(k_{f1}k_{f2}{}^{2}k_{f4}-k_{f1}k_{f2}k_{f4}{}^{2}\right)}{8\left(k_{f1}+k_{f2}-k_{f4}\right)}\right)cos\left[\left(k_{f1}+k_{f2}-k_{f4}\right)\left(x-ct\right)\right]x\\ +\left(\frac{\gamma A_{1}A_{2}A_{3}\left(k_{f1}{}^{2}k_{f2}k_{f3}-k_{f1}k_{f2}{}^{2}k_{f3}-k_{f1}k_{f2}k_{f3}{}^{2}\right)}{8\left(k_{f2}+k_{f3}-k_{f1}\right)}\right)sin\left[\left(k_{f2}+k_{f3}-k_{f1}\right)\left(x-ct\right)\right]x\\ +\left(\frac{\gamma A_{1}A_{2}A_{4}\left(k_{f1}k_{f3}{}^{2}k_{f4}-k_{f1}{}^{2}k_{f3}k_{f4}-k_{f1}k_{f2}k_{f3}{}^{2}\right)}{8\left(k_{f1}+k_{f4}-k_{f3}\right)}\right)sin\left[\left(k_{f1}+k_{f4}-k_{f3}\right)\left(x-ct\right)\right]x\\ +\left(\frac{\gamma A_{3}A_{4}A_{1}\left(k_{f1}{}^{2}k_{f3}k_{f4}-k_{f1}k_{f3}{}^{2}k_{f4}-k_{f1}k_{f2}k_{f4}{}^{2}\right)}{8\left(k_{f3}+k_{f4}-k_{f1}\right)}\right)sin\left[\left(k_{f3}+k_{f4}-k_{f1}\right)\left(x-ct\right)\right]x\\ -\left(\frac{\gamma A_{1}A_{2}A_{4}\left(k_{f1}k_{f2}{}^{2}k_{f4}+k_{f1}k_{f2}k_{f4}{}^{2}\right)}{8\left(k_{f2}+k_{f4}-k_{f1}\right)}\right)cos\left[\left(k_{f2}+k_{f4}-k_{f1}\right)\left(x-ct\right)\right]x\\ +\left(\frac{\gamma A_{1}A_{2}A_{3}\left(k_{f1}k_{f3}k_{f3}{}^{2}-k_{f1}k_{f2}{}^{2}k_{f3}+k_{f1}{}^{2}k_{f2}k_{f3}\right)}{8\left(k_{f1}+k_{f3}-k_{f2}\right)}\right)sin\left[\left(k_{f1}+k_{f3}-k_{f2}\right)\left(x-ct\right)\right]x\\ -\left(\frac{\gamma A_{2}A_{3}A_{4}\left(k_{f2}{}^{2}k_{f3}k_{f4}-k_{f2}k_{f3}k_{f4}{}^{2}\right)}{8\left(k_{f2}+k_{f3}+k_{f4}\right)}\right)cos\left[\left(k_{f2}+k_{f3}+k_{f4}\right)\left(x-ct\right)\right]x\\ -\left(\frac{\gamma A_{2}A_{3}A_{4}\left(k_{f2}{}^{2}k_{f3}k_{f4}-k_{f2}k_{f3}k_{f4}{}^{2}\right)}{8\left(k_{f2}+k_{f4}-k_{f3}\right)}\right)cos\left[\left(k_{f2}+k_{f4}-k_{f3}\right)\left(x-ct\right)\right]x\\ +\left(\frac{\gamma A_{2}A_{3}A_{4}\left(k_{f2}{}^{2}k_{f3}k_{f4}+k_{f2}k_{f3}k_{f4}{}^{2}\right)}{8\left(k_{f2}+k_{f3}-k_{f4}\right)}\right)cos\left[\left(k_{f2}+k_{f3}-k_{f4}\right)\left(x-ct\right)\right]x\\ -\left(\frac{\gamma A_{2}A_{3}A_{4}\left(k_{f2}{}^{2}k_{f3}k_{f4}+k_{f2}k_{f3}k_{f4}{}^{2}\right)}{8\left(k_{f3}+k_{f4}-k_{f2}\right)}\right)cos\left[\left(k_{f3}+k_{f4}-k_{f2}\right)\left(x-ct\right)\right]x\\ -\left(\frac{\gamma A_{1}A_{2}A_{4}k_{f1}k_{f2}k_{f4}{}^{2})}{8\left(k_{f1}+k_{f4}-k_{f2}\right)}\right)cos\left[\left(k_{f1}+k_{f4}-k_{f2}\right)\left(x-ct\right)\right]x. \end{array}$$

## Appendix B

Table A1 summarises the derived nonlinearity parameters with an increasing number of fundamental frequencies. If only one frequency is used, the second and third harmonic frequencies are available for evaluation. With two fundamental frequencies, 12 nonlinearity parameters were derived, including the linear-dependent parameter *δ**f_n_*, the harmonic parameters *δ*2*f_n_*, *δ*3*f_n_*, and the modulated response parameters *δ**f_n_* ± *f_m_* and *δ*2*f_n_* ± *f_m_*. If four frequencies are transmitted in parallel, the number of harmonics and sidebands increases to a maximum of 64. New parameters of the type *δ**f_n_* + *f_m_* ± *f_p_* were added.

The second harmonic parameters *δ*2*f_n_* and sideband *δ**f_n_* ± *f_m_* were derived from the second-order nonlinearity parameter *β*. All others have their origin in the third-order nonlinearity parameter, *γ*.

**Table A1:** Comparison—nonlinearity parameters.

One Excitation Frequency		Two Excitation Frequencies		Four Excitation Frequencies	
Frequencies	Number	Frequencies	Number	Frequencies	Number
-	-	*f_n_*	2	*f_n_*	4
2*f_n_*	1	2*f_n_*	2	2*f_n_*	4
3*f_n_*	1	3*f_n_*	2	3*f_n_*	4
-	-	*f_n_* ± *f_m_*	2	*f_n_* ± *f_m_*	12
-	-	2*f_n_* ± *f_m_*	4	2*f_n_* ± *f_m_*	24
-	-	-	-	*f_n_* + *f_m_* ± *f_p_*	16
	∑2		∑12		∑64
